# Rhizosphere microbiomes diverge among *Populus trichocarpa* plant-host genotypes and chemotypes, but it depends on soil origin

**DOI:** 10.1186/s40168-019-0668-8

**Published:** 2019-05-18

**Authors:** Allison M. Veach, Reese Morris, Daniel Z. Yip, Zamin K. Yang, Nancy L. Engle, Melissa A. Cregger, Timothy J. Tschaplinski, Christopher W. Schadt

**Affiliations:** 1 Biosciences Division, Oak Ridge National Laboratory, 1 Bethel Valley Rd, Oak Ridge, TN 37831-6038 USA; 2 Department of Microbiology, University of Tennessee, Knoxville, TN 37996 USA

**Keywords:** Metabolomics, Salicylic acid, *Populus trichocarpa*, 16S rRNA, ITS2, Rhizosphere

## Abstract

**Background:**

Plants have developed defense strategies for phytopathogen and herbivore protection via coordinated metabolic mechanisms. Low-molecular weight metabolites produced within plant tissues, such as salicylic acid, represent one such mechanism which likely mediates plant – microbe interactions above and below ground. Salicylic acid is a ubiquitous phytohormone at low levels in most plants, yet are concentrated defense compounds in *Populus*, likely acting as a selective filter for rhizosphere microbiomes. We propagated twelve *Populus trichocarpa* genotypes which varied an order of magnitude in salicylic acid (SA)-related secondary metabolites, in contrasting soils from two different origins. After four months of growth, plant properties (leaf growth, chlorophyll content, and net photosynthetic rate) and plant root metabolomics specifically targeting SA metabolites were measured via GC-MS. In addition, rhizosphere microbiome composition was measured via Illumina MiSeq sequencing of 16S and ITS2 rRNA-genes.

**Results:**

Soil origin was the primary filter causing divergence in bacterial/archaeal and fungal communities with plant genotype secondarily influential. Both bacterial/archaeal and fungal evenness varied between soil origins and bacterial/archaeal diversity and evenness correlated with at least one SA metabolite (diversity: populin; evenness: total phenolics). The production of individual salicylic acid derivatives that varied by host genotype resulted in compositional differences for bacteria /archaea (tremuloidin) and fungi (salicylic acid) within one soil origin (Clatskanie) whereas soils from Corvallis did not illicit microbial compositional changes due to salicylic acid derivatives. Several dominant bacterial (e.g., *Betaproteobacteria, Acidobacteria, Verrucomicrobia, Chloroflexi, Gemmatimonadete, Firmicutes*) and one fungal phyla (*Mortierellomycota*) also correlated with specific SA secondary metabolites; bacterial phyla exhibited more negative interactions (declining abundance with increasing metabolite concentration) than positive interactions.

**Conclusions:**

These results indicate microbial communities diverge most among soil origin. However, within a soil origin, bacterial/archaeal communities are responsive to plant SA production within greenhouse-based rhizosphere microbiomes. Fungal microbiomes are impacted by root SA-metabolites, but overall to a lesser degree within this experimental context. These results suggest plant defense strategies, such as SA and its secondary metabolites, may partially drive patterns of both bacterial/archaeal and fungal taxa-specific colonization and assembly.

**Electronic supplementary material:**

The online version of this article (10.1186/s40168-019-0668-8) contains supplementary material, which is available to authorized users.

## Background

Plant microbiomes are a major determinant of plant health, productivity [1, 2] and have the potential to improve sustainable agricultural practices through enhanced growth, nutrient use efficiency, and stress tolerance. Recent work demonstrates the specificity of the microbiomes of the root and rhizosphere (soil immediately surrounding the plant root), within plant species or genotypes [3, 4], and the complex interactions between plant hosts and soil microbiota. However, environmental variation spanning large spatial extents, such as edaphic or climatic conditions [5, 6] to smaller-scale interactions via plant-microbe cellular processes, such as plant-mediated chemical signaling [7], may concurrently impact below ground microbiome development and maintenance. For example, Lebeis et al. (2015) [7] demonstrated that although soil type is influential for root microbial community assembly, genetic variation within plants hosts, in this case *Arabidopsis thaliana,* is associated with differential microbial colonization. Furthermore, belowground versus aboveground microbiomes may display differential shifts in response to plant genetic control. Wagner et al., (2013) [8] indicated plant hosts, specifically, *Boechera stricta*, exhibited greater genetic control in aboveground bacterial communities relative to belowground suggesting the importance of environmental heterogeneity in shaping assembly dynamics, particularly for belowground tissues. The relative importance of edaphic conditions versus host selection processes in determining plant microbiome composition, particularly in tree species, has been largely unexplored [9] and may be dependent on the wide range of physiological or genetic differences among or within plant species.

Variation in edaphic conditions select for specific microbial groups. Physico-chemical variables, such as soil pH [10, 11], nutrients [12, 13], texture [14], micro-aggregate structure [15, 16], among other factors, affect either overall composition or functional group prevalence (i.e., beneficial or pathogenic groups) of bacteria and fungi. Soil conditions will not only impact local rhizosphere microbial community assembly, a subset of bulk soil capable of plant colonization [6, 17], but also influence plant health and metabolism (e.g., photosynthate production, below ground carbon allocation). Thus, the interaction between soil conditions and plant-mediated selective pressures on neighboring microbiota is difficult to parse. Regardless of these complex interactions, microbial composition differences have been detected for specific soil types, plant species, and more rarely genotypes within species [4–6, 18, 19], perhaps indicating the importance of multiple habitat filters for rhizosphere microbial communities surrounding plant roots. Environmental filtering hierarchies are commonly recognized as an operative process in community assembly [20, 21]. Pinpointing the relative roles of such filters under the framework of plant-soil-microbe interactions will be essential in developing a predictive understanding of the microbiome’s regulation of plant health and productivity [22].

Salicylic acid (SA) is a common plant phenolic signaling compound which regulates a range of abiotic host responses, such as responses to drought or salt stress [23, 24] and host physiology such as plant growth and development [25, 26]. Additionally, SA is integral in mediating systemic acquired resistance against biotrophic pathogens and has been identified as such in tobacco [27, 28], *Arabidopsis thaliana* [7, 29, 30], and rice [23, 31]. *Arabidopsis* genotypes with a manipulated systemic expression of SA signaling have been shown to have increased population densities of *Pseudomonas* spp*.* [30] and may regulate colonization of root microbiota by specific bacterial families (e.g., enriched *Streptomycetaceae*) [7]. Although SA is ubiquitous in plants, species vary in SA production [32]. While SA effects on the microbiome have been studied in *Arabidopsis*, less is known about *Populus* spp*.,* although *Populus* spp. produce SA at vastly greater concentrations than most plant species [33]. In *Populus*, and other *Salicaceae*, SA and phenolic glycosides act as inducible defense chemicals [34] expressed in response to pathogen presence and may vary with plant genotype and the developmental stage of the tree [35, 36]. Additionally, in other *Populus* species it has been shown that variation in condensed tannins influences litter decomposition [37], fungal endophyte colonization [38], and based on PLFA profiles, Schweitzer et al. (2007 and 2008) [39, 40] showed these may influence soil microbial composition directly. Thus the chemistry of plant tissues may represent a host-induced filter for the microbiome in the plant-soil environment.

Here, we provide empirical data supporting the relative importance of soil origin (a large-scale environmental filter) and plant genotype and chemotype (a fine-scale environmental filter) on the rhizosphere microbiome (archaea, bacteria, fungi) of an ecologically and economically important model species [41], *Populus trichocarpa* (Black Cottonwood). Soils were collected from 2 separate locations and had different nutrient concentrations and soil texture (Additional file 1: Table S1). Our study goals were to not only identify the relative contribution of soil origin and plant genotype in driving microbiome composition, but also if SA and its secondary metabolite derivatives mediate microbial colonization and assembly in *P. trichocarpa* rhizospheres *after* accounting for soil origin differences. Using twelve clonal genotypes varying an order of magnitude in higher-order salicylate production, we hypothesized that (i) soil origin would be the dominant predictor in explaining microbial compositional divergence and (ii) genotype salicylate production, would be a secondary, fine-scale filter, helping to explain divergence in microbial community composition within closely related plant genotypes.

## Results

### Plant metabolomics and plant trait data

**Fig. 1:**
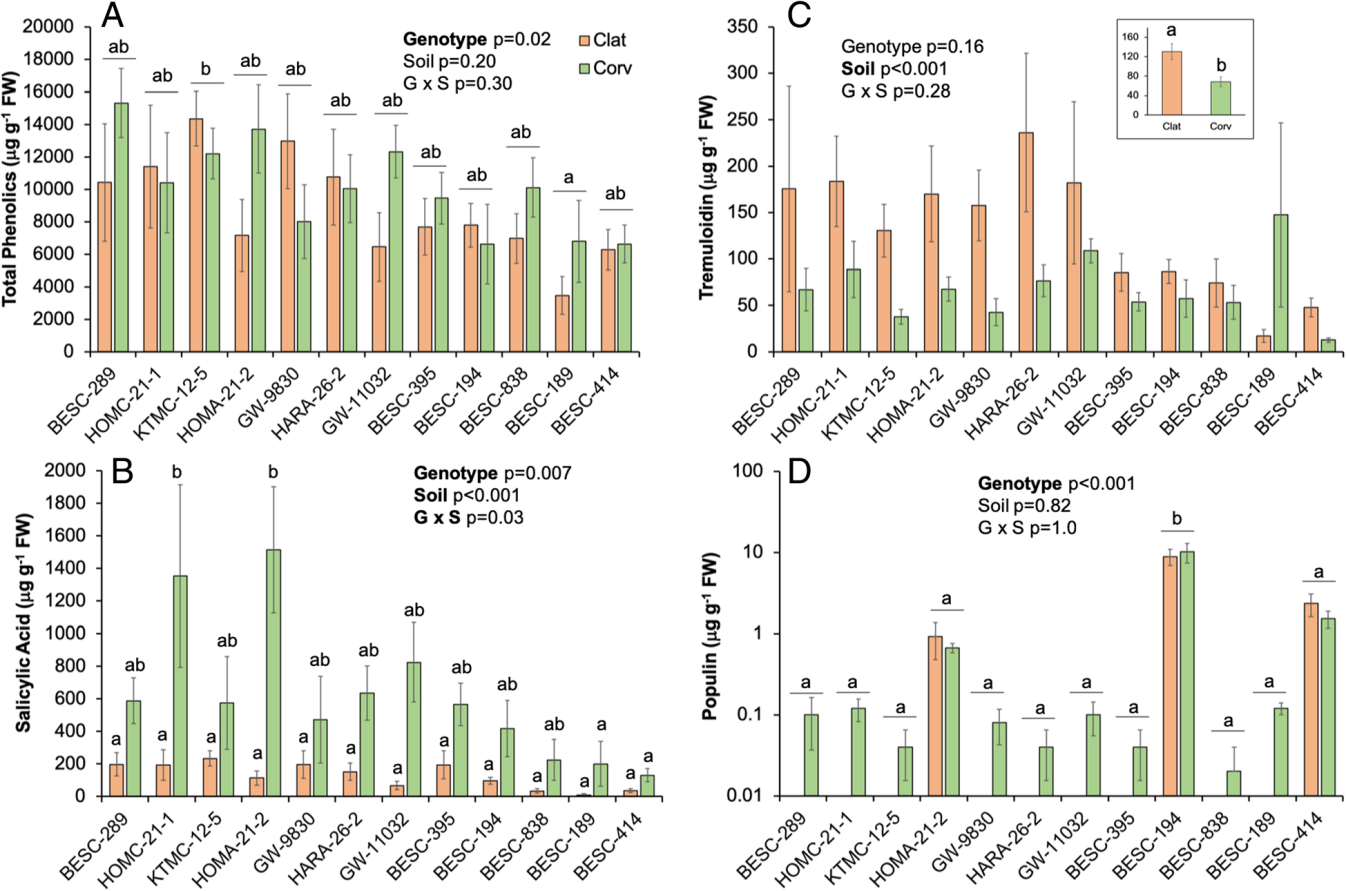
Mean plant metabolites (± standard errors) – total phenolics (Panel **a**), salicylic acid (Panel **b**), tremuloidin (Panel **c**), and populin (Panel **d**) concentrations (μg g^−1^ fresh weight (FW)) in root tissues among genotypes and soil origin. X-axes are ordered based on rank of salicylate concentrations in descending order (BESC-289 > BESC-414). Orange bars denote secondary metabolites from genotypes grown in Clatskanie soils, whereas green bars denote Corvallis soils. Letters denote significant differences calculated from Tukey HSD tests among genotypes and soil origins. Tremuloidin only differed between soil origins therefore additional panel is included representing the mean tremuloidin concentrations across all genotypes grown in Clatskanie versus Corvallis soils (Panel **c**). Note Panel **d** Y-axis is on a logarithmic scale

Among genotypes, mean total phenolics ranged from 5548 to 13,269 μg g FW^−^ 1, salicylic acid ranged from 34 to 1515 μg g FW^− 1^, tremuloidin ranged from 17 to 184 μg g FW^− 1^, and populin ranged from 0.01 – 9.4 μg g FW^− 1^ and varied widely among genotypes (Fig. 1; Additional file 1: Table S2). All metabolites, except salicin and total salicylates, varied among tree genotypes or between soil origins: total phenolics (*p* = 0.02), catechin (*p* < 0.001), a-salicyloylsalicin (*p* = 0.008), salicortin (*p* = 0.007), trichocarpin (*p* = 0.003), and populin (*p* < 0.001) were differentially produced at least within one genotype versus another (Additional file 1: Table S2). Salicylic acid had a significant genotype x soil origin interaction; two genotypes expressed greater salicylic acid concentrations in Corvallis soils specifically to the majority of the other 10 genotypes within both Corvallis and Clatskanie soils (Fig. 1). Furthermore, tremuloidin did not vary among genotypes, but was differentially produced by trees between soil origins (*p* < 0.001; Fig. 1c). On average, plants grown in Clatskanie soils had greater tremuloidin production within roots compared to Corvallis soils (Fig. 1c).

Leaf growth (*p* < 0.01), chlorophyll content (*p* < 0.01), and photosynthetic rate (*p* < 0.01) differed among plant genotype and between soil origin (Additional file 1: Table S3). All of these responses were greater in the nutrient-rich Clatskanie soils versus Corvallis. Genotype effects for plant measurements were due to one genotype displaying a difference between 1 or 2 other genotypes. BESC-395 expressed greater photosynthetic rate compared to BESC-414 and BESC-838 (Tukey’s HSD: *p* ≤ 0.05). Leaf chlorophyll content was lower for BESC-838 compared to GW-9830 and KTMC-12-5 (Tukey’s HSD: *p* < 0.02) whereas BESC-838 had greater leaf growth compared to these two genotypes (Tukey’s HSD: *p* < 0.02). Furthermore, photosynthetic rate (*p* ≤ 0.05) and chlorophyll content (*p* ≤ 0.02) correlated with either catechin, tremuloidin, or salicylic acid, but this relationship depended on soil origin (Additional file 1: Table S4); leaf growth did not correlate with any metabolite regardless of soil origin (*p* ≥ 0.06). Ectomycorrhizal colonization rates of poplar root-tips did not differ between soil origins (*p* = 0.89) or plant genotype (*p* = 0.18) but was negatively correlated with tremuloidin (T = -2.28, *p* = 0.03, Full Model: Adj. R^2^ = 0.07, F_1,56_ = 5.19, *p* = 0.03).

### Bulk soil and rhizosphere compositional differences

In bulk soil microbial communities (e.g. no-plant controls), both bacteria/archaea and fungi, differed in their dominant taxa relative to rhizosphere communities among genotypes. Notably, on average, Clatskanie bulk soils were enriched in *Crenarchaeota* (2.0% relative abundance in soils versus 0.3% in rhizospheres) and *Nitrospirae* (1% in soils, 0.4% in rhizospheres), and depleted in *Firmicutes* (1.3% soils, 2.2% rhizospheres) and *Acidobacteria* (20% soils, 17.6% rhizospheres). Clatskanie soils were also enriched in *Nitrospirae* (1.4% versus 0.8% rhizospheres) and *Verrucomicrobia* (7.7% soils, 5.4% rhizospheres) and depleted in *Firmicutes* (1.8% versus 2.5% rhizospheres) as well as *Actinobacteria* (14% versus 23% rhizospheres) and *Acidobacteria* (15% soil, 12% rhizospheres). For fungi in Clatskanie soils, all phyla exhibited substantial differences in abundance between bulk soils and rhizospheres. *Chytridiomycota* (1.9% soils, 0.7% rhizospheres) and *Mortierellomycota* (9% soils, 4% rhizospheres) were enriched in soils and *Ascomycota* (20% soils, 25% rhizospheres), *Basidiomycota* (36% soils, 56% rhizospheres), and *Glomeromycota* (3% soils, 7% rhizospheres) were depleted in soils relative to rhizospheres. However, in Corvallis soils, only *Glomeromycota* showed substantial turnover between these comparments: 0.4% on average in soils versus 1.3% in rhizospheres.

### Microbial alpha diversity

**Fig. 2:**
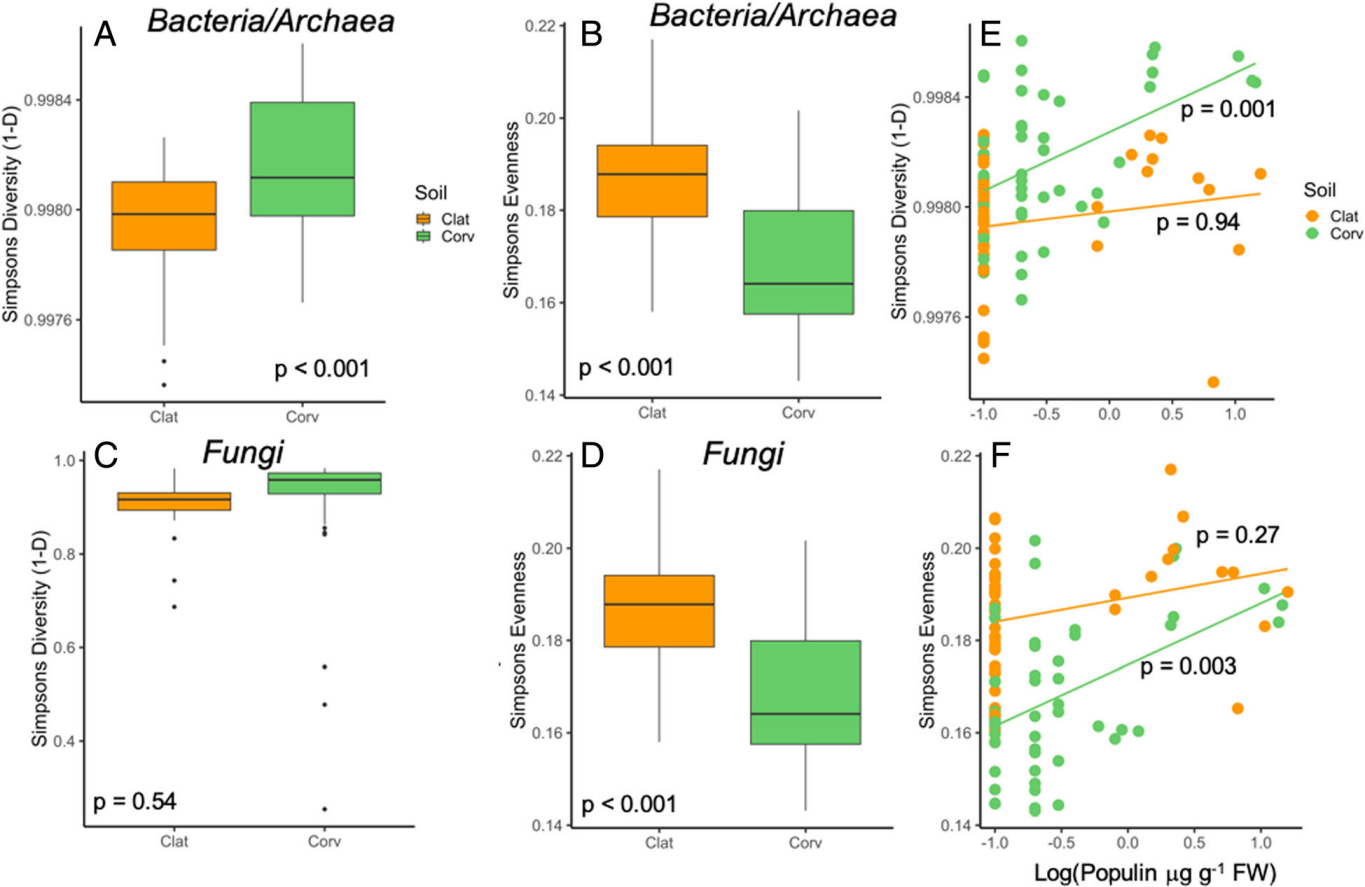
Bacterial/archaeal and fungal diversity (Simpson’s Diversity: 1-D; Panel **a**, **c**) and Simpsons’s Evenness ( Panel **b**, **d**) in Clatskanie and Corvallis soil origins. Orange boxplots and points denote Clatskanie and green denotes Corvallis soils. Bacterial/archeal diversity and evenness was correlated with populin concentration in Corvallis soils (Panel **e**, **f**). Type-1 error rates given were generated by stepwise regression model analyses

Bacterial/archaeal Simpson’s Diversity and Evenness differed among soil origins, plant genotype, and there were significant interactions between soil origin and plant genotype (Table 1, Additional file 1: Figure S1). Bacterial/archaeal diversity was 0.02% greater in Corvallis soils, whereas evenness was ~ 10% greater in Clatskanie soils (Fig. 2a). Fungal diversity did not differ between soil origins (*p* > 0.50). Fungal evenness did not differ among genotypes, but contrary to bacterial/archaeal evenness, it was ~ 54% greater in Corvallis soils compared to Clatskanie and had a significant soil origin x genotype interaction (Table 1, Fig. 2d, Additional file 1: Figure S1). Bacterial/archaeal diversity and evenness both correlated with specific metabolites in the nutrient-poor Corvallis soils: diversity and evenness increased with populin concentrations (respectively: T = 2.76, Full Model Adj R^2^ = 0.11, *p* = 0.01; T = 3.52, Full Model Adj. R^2^ = 0.21, *p* < 0.01; Fig. 2e,f) whereas evenness also increased with total phenolics (T = 2.40, *p* = 0.02) and declined with increasing tremuloidin production (T = − 2.24, *p* = 0.03).

**Table 1:** Two-way ANOVA model summary for responses of Simpson’s Diversity and Evenness for bacteria/archaeal and fungal communities and explanatory variables of soil origin, genotype, and their interaction. Explanatory variables deemed statistically significant are bolded

Response variable	Explanatory Variable	DF	F-value	*p*-value
Bacterial/Archaeal Diversity	**Soil Origin**	**1**	**16.23**	**< 0.01**
	**Genotype**	**11**	**3.05**	**< 0.01**
	**Interaction**	**11**	**2.22**	**0.02**
Bacterial/Archaeal Evenness	**Soil Origin**	**1**	**50.63**	**< 0.01**
	**Genotype**	**11**	**4.22**	**< 0.01**
	**Interaction**	**11**	**2.34**	**0.02**
Fungal Diversity	Soil Origin	1	0.39	0.54
	Genotype	11	0.77	0.67
	Interaction	11	1.03	0.43
Fungal Evenness	**Soil Origin**	**1**	**22.04**	**< 0.01**
	Genotype	11	1.16	0.32
	**Interaction**	**11**	**2.08**	**0.03**

### Microbial beta-diversity

**Fig. 3:**
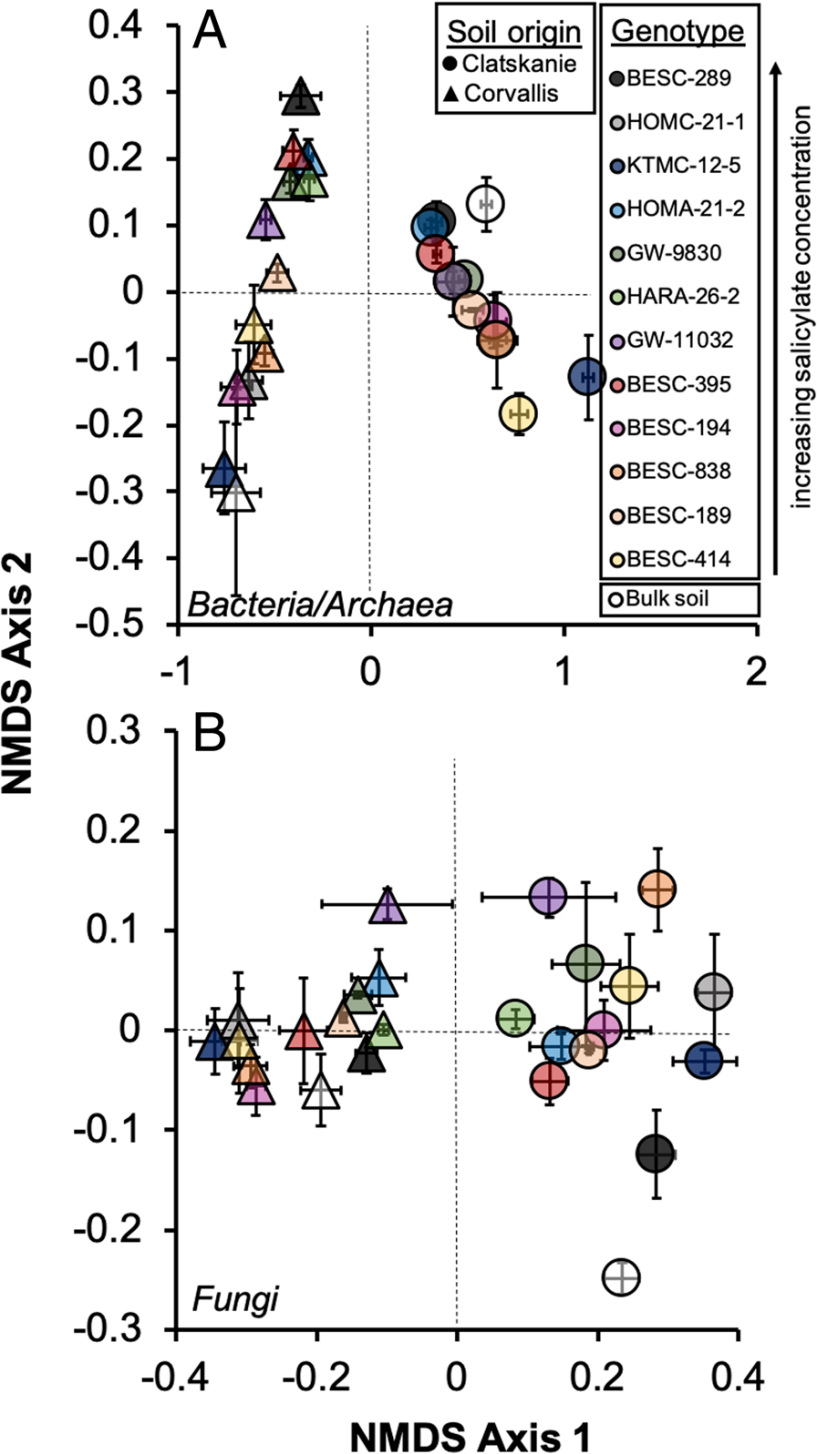
Non-metric dimensional scaling ordination for bacteria/archaea (**a**) and fungi (**b**) among twelve genotypes and between Clatskanie and Corvallis soil origins. Points represent mean ordination scores (i.e., the centroid, ± standard errors) with colors denoting genotype and shape denoting soil origin. Circles represent microbial communities grown in Clatskanie soils and triangles represent Corvallis soils. Soil origin and genotype explained a large proportion of variation in community composition for both bacteria/archaea (~ 59%) and fungi (~ 45%; Table 3). Stress scores were ~ 0.06 for both bacteria/archaea and fungal community ordinations

In congruence with our hypotheses, soil origin accounts for a large proportion of variation in OTU-level composition for both bacteria/archaea (R^2^ = 0.47) and fungi (R^2^ = 0.33; Table 3, Fig. 3), with genotype secondarily influential and accounting for ~ 10-12% of variation in bacteria/archaea and fungi communities respectively (Table 3, Fig. 3). For both bacteria/archaea and fungal community composition, the interaction between soil origin and genotype was significant (Table 3) and explained an additional 8-9% of compositional variation (Table 3, Fig. 3). Specifically, for both bacteria/archaea (*p* = 0.08) and fungal communities (*p* = 0.15), genotype GW-11032 did not exhibit significant shifts in composition between soil origins; for bacterial/archaeal communities only, genotype HARA-26-2 also did not exhibit a significant shift in composition between soil origins (*p* = 0.11). All other genotypes exhibited significant differences in composition for bacteria/archaea and fungi between Clatskanie and Corvallis soils (FDR-correction: *p* < 0.01; Fig. 3). Bulk soils also differed in composition for bacteria/archaea and fungi (Fig. 3). Lastly, both bacterial/archaeal and fungal communities were influenced by one SA-secondary derivative in Clatskanie soil (CAP model: *p* < 0.05) but were not affected by these in Corvallis soils (CAP model: *p* > 0.20). Specifically, Clatskanie bacteria/archaea community composition was influenced by tremuloidin (F_1,39_ = 3.17, *p* = 0.003) and salicortin concentrations (F_1,39_ = 1.88, *p* = 0.03) within roots. Fungal community composition in Clatskanie soils was influenced by salicylic acid (F_1,43_ = 2.13, *p* = 0.006).

### Dominant microbial taxa shifts

**Fig. 4:**
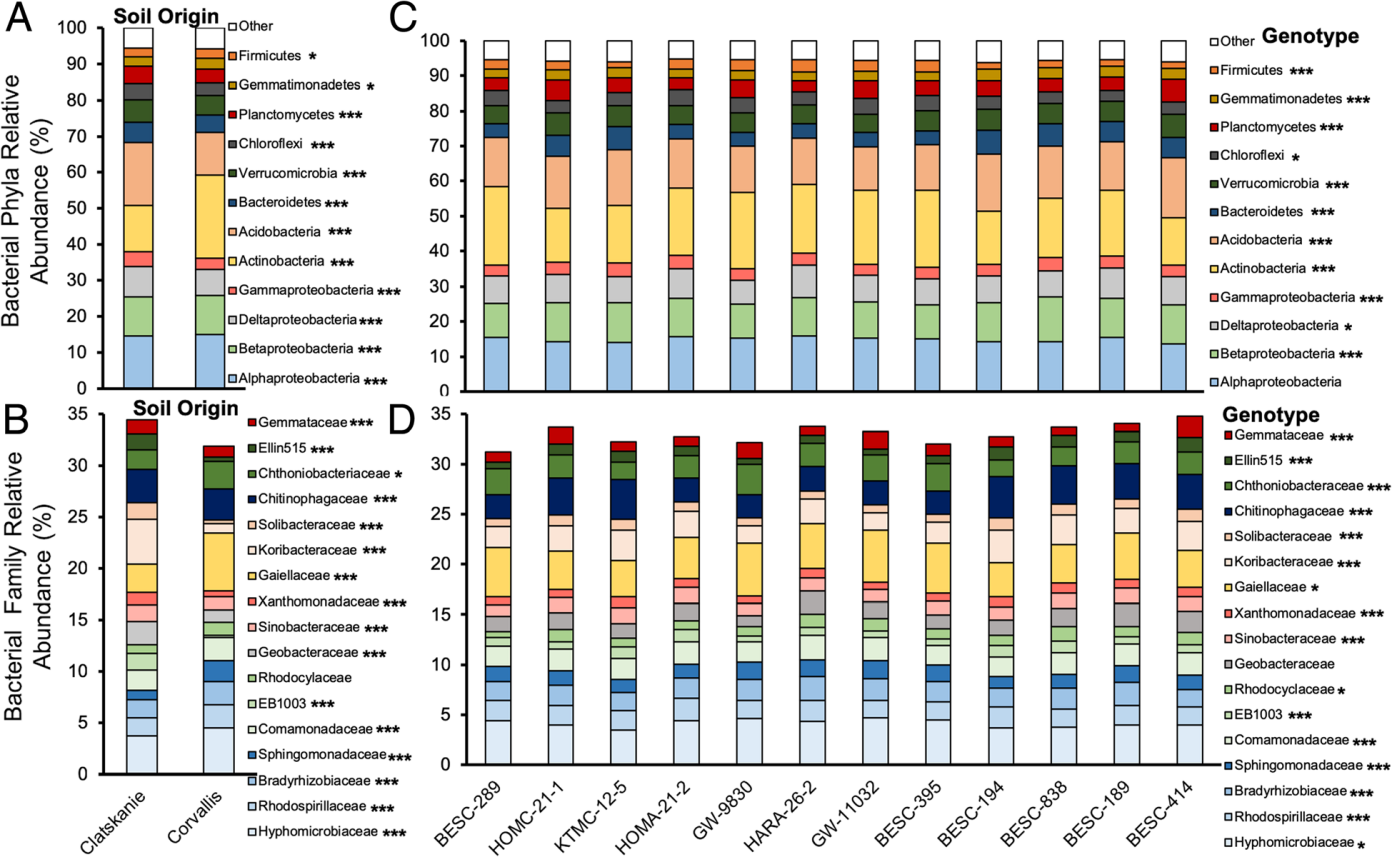
The relative abundance of dominant bacterial phyla (class for Proteobacteria) and families within soil origins (Panel **a**, **b**) and among genotypes (Panel **c**, **d**). Asterisks denote significant differences in abundance between soil origins or genotype generated by two-way ANOVA models and with an FDR-statistical correction applied. Raw counts were centered log-ratio transformed prior to ANOVA models. Due to a large portion of reads belonging to non-dominant families (> 50% all reads), an “Other” category is used for clarity

**Fig. 5:**
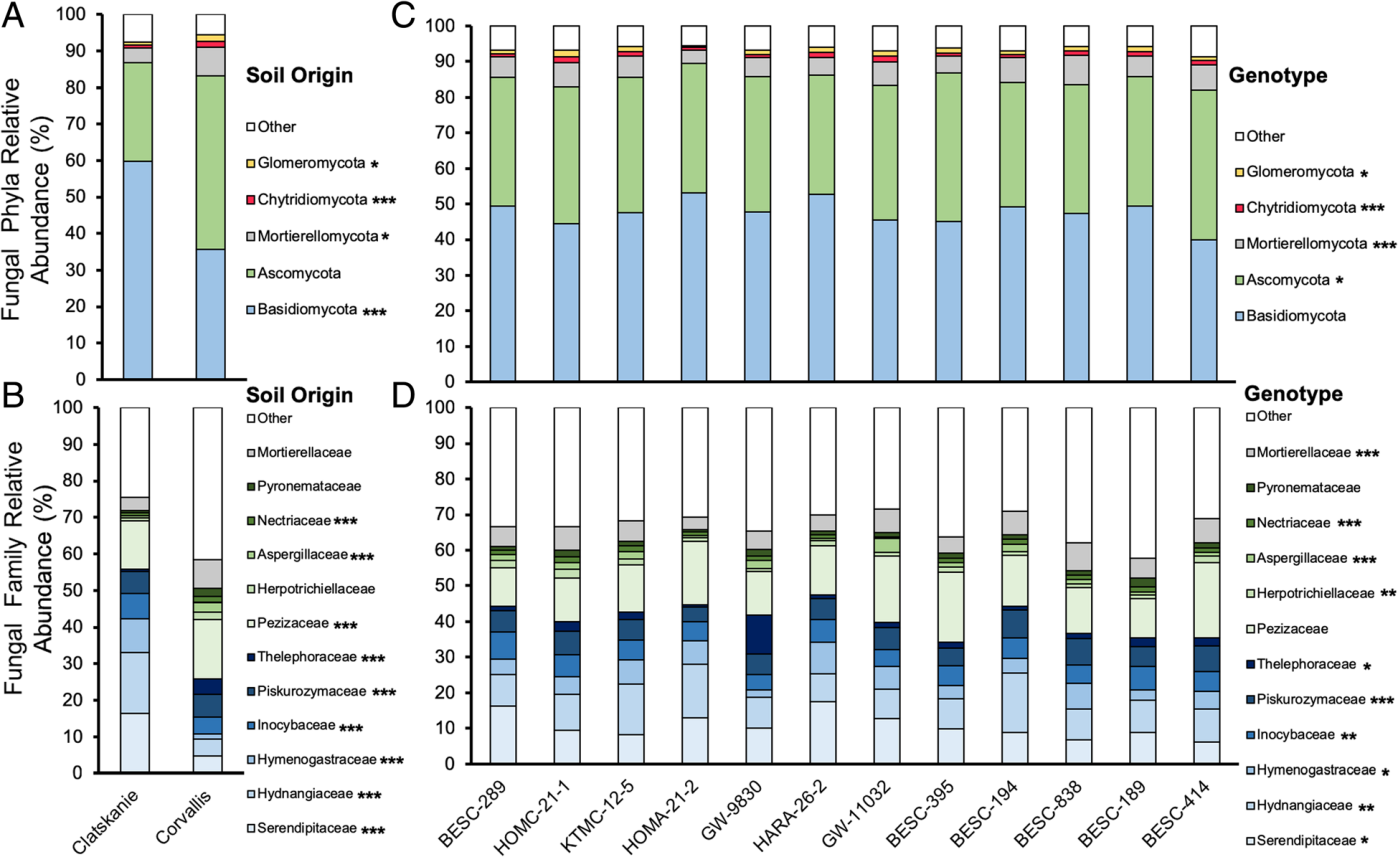
The relative abundance of dominant fungal phyla and families within soil origins (Panel **a**, **b**) and among genotypes (Panel **c**, **d**). Asterisks denote significant differences in abundance between soil origins or genotype generated by two-way ANOVA models and with an FDR-statistical correction applied. Raw counts were centered log-ratio transformed prior to ANOVA models. Due to a large portion of reads belonging to non-dominant families an “Other” category is used for clarity

Out of twelve dominant (> 1.0% relative abundance) bacterial phyla, all differed between soil origins and among plant genotypes, except *Alphaproteobacteria* which did not vary among genotypes (Fig. 4c, Additional file 1: Table S5). Out of 5 dominant fungal phyla, all except *Ascomycota* differed between soil origin, and all except *Basidiomycota* differed among genotypes (Fig. 5c, Additional file 1: Table S6). Clatskanie soils had significantly lower abundances of *Actinobacteria*, but greater abundances of *Acidobacteria* (Fig. 4). Furthermore, Clatskanie soils had significantly greater *Basidiomycota* and lower abundances of other dominant fungal phyla (*Ascomycota*, *Mortierellomycota*, *Chytridiomycota*, and *Glomeromycota;* Fig. 5). All seventeen dominant bacterial families differed in abundance between soil origins (Fig. 3b) and among genotypes except *Geobacteraceae* (Fig. 4d, Additional file 1: Table S5). Nine out of 12 dominant fungal families differed between soil origins whereas ten differed among plant genotypes (Fig. 4, Additional file 1: Table S6).

All bacterial phyla either correlated with a secondary metabolite or between soil origin (Table 2). *Betaproteobacteria*, *Acidobacteria*, *Verrucomicrobia*, *Chloroflexi*, and *Gemmatimonadetes* correlated with salicylic acid and/or populin (Table 2)*.* Generally, these phyla declined in abundance with increasing SA concentrations, but increased with populin concentrations (Table 2), except *Chloroflexi* exhibited a positive correlation with SA. In addition, *Actinobacteria* and *Firmicutes* positively correlated with tremuloidin; *Firmicutes* also correlated with catechin and both *Firmicutes* and *Deltaproteobacteria* negatively correlated with total phenolics (Table 2). Several bacterial families also correlated with salicylic acid or populin (Bacteroidetes *Chitinophagaceae,* Acidobacterial *Koribacteraceae* and *Solibacteraceae,* Deltaproteobacterial *Geobacteraceae,* Verrucomicrobial *Ellin515,* and Betaproteobacterial *Rhodocyclaceae*; Additional file 1: Table S7). One fungal phylum varied with secondary metabolites: *Mortierellomycota* correlated with 2 metabolites: negative correlation with tremuloidin and a positive correlation with populin (Table 2). Four out of 12 fungal families correlated with specific secondary metabolites (Additional file 1: Table S7). The basidiomycete *Hymenogastraceae* and likely saprobic *Mortierellaceae* correlated with SA, although *Hymenogastraceae* increased in abundance with SA, whereas *Mortierellaceae* declined in abundance with SA. The basidiomycete family *Piskurozymaceae* positively correlated with populin, and ascomycete family *Nectriaceae* negatively correlated with tremuloidin (Additional file 1: Table S7).

**Table 2:** Multiple stepwise regression model results for dominant bacterial and fungal phyla (and classes for Proteobacteria) that significantly correlated with a secondary metabolites. Soil origin was also included as an explanatory variable to discern metabolite versus soil effects. Raw abundances were centered log-ratio transformed prior to analysis. Type-1 error rates given are FDR-corrected

				Full model statistics		
Microbial Phylum	Explanatory variable	T	*p-value*	F-statistic	Adj. R^2^	*p*-value
Bacteria						
*Betaproteobacteria*	Intercept	67.3	< 0.01	13.07	0.19	< 0.01
	**Soil(Corvallis)**	**−2.84**	**< 0.01**			
	**salicylic acid**	**−2.67**	**< 0.01**			
*Deltaproteobacteria*	Intercept	17.99	< 0.01	21.99	0.38	< 0.01
	**Soil(Corvallis)**	**−7.33**	**< 0.01**			
	catechin	1.74	0.09			
	**phenolics**	**−2.17**	**0.03**			
*Acidobacteria*	Intercept	70.39	< 0.01	51.94	0.6	< 0.01
	**Soil(Corvallis)**	**−10.26**	**< 0.01**			
	saliyclic acid	−1.49	0.14			
	**populin**	**2.66**	**< 0.01**			
*Actinobacteria*	Intercept	45.92	< 0.01	25.79	0.42	< 0.01
	**Soil(Corvallis)**	**8.48**	**< 0.01**			
	**tremuloidin**	**2.25**	**0.03**			
	populin	−1.85	0.07			
*Verrucomicrobia*	Intercept	62.91	< 0.01	22.79	0.39	< 0.01
	**Soil(Corvallis)**	**−5.68**	**< 0.01**			
	**salicylic acid**	**−2.56**	**0.01**			
	**populin**	**2.29**	**0.02**			
*Bacteroidetes*	Intercept	36.34	< 0.01	10.82	0.22	< 0.01
	**Soil(Corvallis)**	**−3.49**	**< 0.01**			
	salicylic acid	−1.82	0.07			
	**populin**	**2.51**	**0.01**			
*Chloroflexi*	Intercept	48.68	< 0.01	41.9	0.45	< 0.01
	**Soil(Corvallis)**	**−8.98**	**< 0.01**			
	**salicylic acid**	**2.15**	**0.03**			
*Gemmatimonadetes*	Intercept	45.06	< 0.01	7.65	0.12	< 0.01
	**salicylic acid**	**−2.79**	**< 0.01**			
	**populin**	**2.75**	**< 0.01**			
*Firmicutes*	Intercept	7.93	< 0.01	3	0.08	0.02
	**tremuloidin**	**2.93**	**< 0.01**			
	**catechin**	**2.24**	**0.03**			
	populin	−1.53	0.13			
	**phenolics**	**−2.11**	**0.04**			
Fungi						
*Mortierellomycota*	Intercept	24.9	< 0.01	5.96	0.09	< 0.01
	**tremuloidin**	**−2.42**	**0.02**			
	**populin**	**2.41**	**0.02**			

Explanatory variables deemed statistically significant are bold

## Discussion

In this study, we provide evidence that (1) soil origin representative of either abiotic differences and/or overall divergence in regional species pools causes plant associated microbial compositional differences; (2) after accounting for soil effects, plant genotype and to some degree, chemotype, acts as a selective pressure in structuring belowground microbial communities, particularly bacterial taxa. Although plant genotypes varied substantially in overall SA and SA-derivative production (Fig. 1), an established gradient of root metabolites was observed and selected for specific microbial groups in the rhizosphere, but these responses varied by soil origin. Furthermore, secondary metabolite production did exhibit plasticity. Several compounds had an interaction with soil origin (salicylic acid, tremuloidin; Fig. 1c) indicative of the impact of environmental selection on host physiology. Regardless of soil effects on metabolite production, archaeal and bacterial taxa and to a lesser degree fungal taxa, did vary not only based on soil origin and host-genotype, but also by specific metabolites, such as SA and populin (Fig. 2) and tremuloidin (Fig. 3). Fungal community composition had more unexplained variance relative to SA profiles, not only in terms of alpha-diversity, but also for tax on abundances: as several phyla did not differentially change in response to metabolites. These data indicate that bacterial communities are relatively more responsive to salicylic acid and its derivatives in belowground *Populus*-associated microbial communities. These outlined responses may point towards two potential mechanisms: (i) soil nutrient status or other physico-chemical variables which vary between soil type drive how microbial communities respond to host-secondary metabolome in roots; or (ii) the microbial taxa comprising regional species pools may differ taxonomically and also in functional trait expression, and therefore respond differentially to plant chemical signaling in the rhizosphere.

Compared to plant genotype and chemotype, soil origin was the primary habitat filter which resulted in microbial community divergence belowground (~ 47% explained community variation for bacteria/archaea, ~ 33% for fungi; Table 3, Fig. 3). This is a consistent finding with other studies in the *Populus* root-rhizosphere microbiome [4, 42] and other plant species [7, 8, 43, 44], indicating larger-scale edaphic conditions primarily regulate overall soil microbiomes and those available for rhizosphere colonization, under most certain contexts. Our greenhouse growth conditions maintained the same rate of water supply and similar environmental conditions; thus, plant growth and microbial community differences were due to soil-specific factors. For example, Colin et al., 2017 [44] indicate that across a toposequence which varies in soil nutrient status, beech trees selectively recruit microbial taxa near roots dependent on soil conditions. This effect is due to environmental conditions regulating plant host metabolism, and therefore will result in strong plant-microbe interactions. In this study, genotypes grown in Clatskanie soils had greater aboveground leaf growth, chlorophyll content, and net photosynthetic rates (Additional file 1: Table S3) likely due to greater nutrient content [45] although other variables related to soil quality may have also cause increased plant growth. For example, Corvallis soils have a finer soil texture and lower nitrogen content, whereas Clatskanie soils were primarily composed of clay-sized particles (Additional file 1: Table S1). These initial differences in nutrient status may have been further impacted by water retention and possible nutrient leaching over the course of the 4 month greenhouse experiment [46]. In addition to these physico-chemical differences in soils, bulk soils (incubated without plants during this study) exhibit large differences in microbial communities among soil origin and differed in microbial composition relative to most genotype rhizospheres (Fig. 3). This effect was particularly heightened for fungal communities in the nutrient-rich Clatskanie soils. Notably, the ratio of basidiomycetes to ascomycetes is quite striking between soil origin: Clatskanie soils have much higher proportions of basidiomycetes, and ectomycorrhizal basidiomycetous families, such as *Hydnangiaceae*, and general mycorrhizal or endophytic families, such as *Serendipitaceae*. Clatskanie soils also have much lower abundances of *Actinobacteria*, and notably the novel family of *Gaiellaceae*, which have been suggested to be associated with plants [47]. Furthermore, Clatskanie bulk soils had a greater proportion of *Acidobacteria* than Corvallis bulk soils (Fig. 4) and likewise, rhizosphere communities in Clatskanie incubations had enrichment of *Acidobacteria* compared to Corvallis. Such results suggest that species pools were substantially different among soil origins, which likely had a strong impact on functionality of the microbiome. However, for fungal communities, some groups, such as *Chytridiomycota* and *Glomeromycota* had similar proportions between soil origins, but plants grown in these soils had differential recruitment of these groups. *Chytridiomycota* was 1.9% on average in Clatskanie and Corvallis bulk soils yet, Corvallis soils had a greater recruitment of this group (1.2% rhizospheres) relative to Clatskanie (0.7% rhizospheres). Such results indicate that not only are regional species pool differences impactful in colonization and assembly of microbes, but also highlights the importance of plant selective processes in structuring the rhizosphere microbial community. Therefore, differences in environmental conditions, regional pools of microbial communities specific to a soil-type, and plant selection may ultimately regulate plant-host microbiome composition and subsequently host health.

**Table 3:** Permutational Multivariate ANOVA results using Euclidean distance matrices for bacterial/archaeal and fungal communities and soil origin (Corvallis, Clatskanie soil), genotype, and their interaction. Raw OTU counts were centered log-ratio transformed prior to Euclidean distance calculations. 999 permutations were used to calculate significance values

Community	Source of variation	R^2^	Pseudo-F	*p*-value
*Bacteria/Archaea*	**Soil Origin**	**0.47**	**113.56**	**0.001**
	**Genotype**	**0.12**	**2.69**	**0.001**
	**SxG Interaction**	**0.08**	**1.66**	**0.006**
	Residuals	0.33		
*Fungi*	**Soil Origin**	**0.33**	**59.73**	**0.001**
	**Genotype**	**0.12**	**1.95**	**0.001**
	**SxG Interaction**	**0.09**	**1.57**	**0.007**
	Residuals	0.46		

Explanatory variables deemed statistically significant are bold

Although soil type was the main driver of microbiome composition, specific SA-derivatives regulated microbial colonization and assembly and the specific derivatives that had effects that were different between soil origins and for bacteria/archaea versus fungi under certain contexts. Bacterial alpha and beta diversity correlated with populin (Fig. 2) whereas fungal evenness correlated total with phenolics and tremuloidin. However, the majority of dominant taxa correlated with SA, populin (*Betaproteobacteria*, *Acidobacteria*, *Verrucomicrobia*, *Mortierellomycota*, etc.) or tremuloidin (*Firmicutes*, *Mortierellomycota*)– this was demonstrated for both bacteria and fungi. Lebeis et al., 2015 [7] demonstrated that isogenic *A. thaliana* mutants lacking SA signaling caused significant changes to root endophyte bacterial communities, notably depletion of *Firmicutes*, and specific classes of *Proteobacteria* (Alpha-, Beta-), which was mirrored at higher taxonomic resolutions (family and OTU-level). This study differs compared to Lebeis et al., (2015) [7] in that SA is constitutively produced among all genotypes of study representing a chemical gradient (low to high concentrations) rather than presence/absence of the smaller hormonal levels of SA in Arabidopsis [7]. Similar to other studies, we found that specific bacterial phyla and fungal families (Figs. 4, 5) respond to SA-derivatives, but most effects occurred in response to only specific derivatives and were multi-directional (positive or negative interactions as indicated by regression model beta coefficients). SA signaling pathways are regulated partially for systemic-acquired resistance to phytopathogens and are typically studied in light of pathogen infection [48], yet we see that diverse microbial community’s representative of natural settings may respond differentially to SA. These variable responses in the rhizosphere suggest that not only does SA production strongly select for specific microbiota, but microorganisms have ecological strategies to tolerate (neutral interaction) or even metabolize specific compounds (positive interaction) [7, 49]. For example, bacterial communities in gypsy moth midguts metabolize phenolic glycosides and reduce plant defense chemicals [50]. Zhalnina et al., 2018 [41] demonstrated that rhizosphere bacteria exhibit nutritive preferences for specific plant exudate organic acids, such as salicylic acid, therefore it is plausible that such secondary metabolites are beneficially regulating rhizosphere microbiome composition. This is further evidenced by several microbial groups exhibiting positive interactions with SA and other derivatives, such as *Chloroflexi* and the basidiomycete family *Hymenogastraceae*, increasing in abundance with SA. However, further evidence is required to validate such an assertion as turnover in microbial taxa may also be impacted by competitive interactions among groups.

Many bacterial taxa responded to genotype and chemotype (SA-derivatives) with varying interactions specific to different taxonomic groups, whereas less fungal taxa responded to host genotype and chemotype although *Mortierellaceae* and *Hymenogastraceae* did correlate with variation in metabolites among genotypes. Ectomycorrhizal colonization rates were not impacted by soil origin, plant genotype, or the majority of secondary metabolites (although did correlate with tremuloidin to some degree) demonstrating the relative differences in microbial response magnitudes of bacteria versus fungi to our experimental manipulation. High SA levels have been linked to either a delayed AM fungal colonization [51] or even the inability for specific AMF species to colonize [52, 53], indicating that SA signaling impacts not only pathogenic infection, but also may inhibit mutualistic interactions specifically with AMF. Our data suggest that such patterns do not extend to all mycorrhizal species as we did not see any differences in ECM colonization and there were actually increases in the *Hymenogastraceae* with SA concentrations in our amplicon-based analyses. Pfabel et al., 2012 [54] found that although SA production was greater in poplars infected with the fungal rust, *Melampsora larici-populina*, there was no interaction between ECM colonization and SA. However, it is plausible that microbial feedbacks with plant hosts, such as mycorrhization, can directly reduce plant host disease resistance responses via SA production [47], but we cannot discern in this study the effective mechanism for plant SA levels and whether microbiome types may regulate its production.

## Conclusions

Plant – soil – microbe interactions and the role of plant secondary metabolism via SA are largely unexplored in relation to diverse, exogenous microbiomes. This study confirms the importance of large-scale conditions and environmental heterogeneity on driving soil microbiome assembly, but additionally validates the contribution of plant host genotype and chemotype in acting as a selective pressure in the surrounding rhizosphere soil. Specifically, levels of SA and its derivatives appear to result in shifts of key bacterial/archaeal and fungal groups in the rhizosphere within differing soil origins. Initiatives using *Populus* as a bioenergy stock may need to consider not only the interplay between genotype and the belowground microbiome, but also the host chemotype which can vary substantially among and within genotypes. These results should be a key consideration for future plant – microbial interactions research attempting to integrate plant metabolomes and microbiomes.

## Methods

We propagated cuttings in climate-controlled greenhouse settings to detect differences in rhizosphere microbial communities among genotypes and between soil origin under otherwise identical conditions. The cuttings originated from 2 to 3 clonal replicates of each tree genotype from a field-grown common garden *Populus trichocarpa* population that has been maintained since 2009 in Corvallis, Oregon. The common garden spans 120 × 150 m with three replicate blocks so three replicates all are subjected to roughly equivalent soil and climate conditions. Out of these ~ 1100 genotypes in the population, 851 genotypes’ leaf tissues were previously analyzed for salicylic acid and higher-order SA conjugate profiles, including salicortin (see methods below). From these data, twelve genotypes that varied by orders of magnitude of in overall SA production and the concentration of various SA derivatives, including salicin and salicortin, were selected for greenhouse experiments. Our preliminary data on a subset of approximately 30 genotypes showed leaf salicortin concentrations were highly correlated with root concentrations in these populations (regression R^2^ = 0.93; unpublished data).

### Plant and soil collections

In January of 2016, 15-20 dormant cuttings were collected from each of 12 clonal genotypes (approximately 200 cuttings in total) of *Populus trichocarpa* at a common garden near Corvallis, Oregon. Cuttings were kept on ice, shipped overnight, and maintained at 4 °C until rooting took place in February. Field soils (top 10 cm) were used as a primary microbial inoculum at time of planting (after rooting took place) in greenhouse settings and were collected from two sites in Oregon – a common garden location adjacent to the Willamette River (referred to as Corvallis soils) and a replicate common garden from a nutrient-rich floodplain approximately 175 km north adjacent to the Columbia River (referred to as Clatskanie). Samples from each site were made from six mini-pits dug in areas adjacent to and between rows of the *Populus* plantation at each site. Soils were shipped on ice overnight and maintained at 4 °C until plants were transplanted after taking root in March 2016. Soils from each site were composited, homogenized, and allowed to air dry for 3 days to a similar water contents prior to the experiment. The soils at these sites had been previously characterized for %OM, %C, %N and soil texture in 2012 (Additional file 1: Table S1) at the University of Georgia Agricultural and Environmental Services Laboratories (http://aesl.ces.uga.edu/).

### Greenhouse experimental design and plant measurements

Each soil (Clatskanie and Corvallis) was mixed with sterile sand in a 2:1 (soil: sand) mixture, to allow for adequate drainage during the greenhouse experiment. Cuttings were rinsed with DI water and a 1% Zerotol 2.0 solution for surface sterilization and placed in sterile sand with rooting powder (0.1% indole-3-butyric acid) at the cutting base to elicit root growth. Once significant root growth was evident among genotypes (~ 6 weeks), 10 replicate cuttings per genotype (120 plants) were transplanted to 3-L pots and the soil:sand mixture described above. Half of these were transplanted in Corvallis soils, the other half transplanted in Clatskanie soils for a total of 5 replicates per genotype within each soil origin. In addition, bulk soils controls of each soil:sand mixture were potted with no plants, were included and treated the same as the pots with experimental plants throughout the growth period in the greenhouse. A drip irrigation system connected to a DI water source was set up to irrigate both planted pots and bulk soil controls every 12 h for 10 min to prevent drought stress. Plants were allowed to grow for ~ 4 months. Approximately 2 weeks prior to harvest, plants were measured for leaf chlorophyll content via a SPAD-502 Meter (Spectrum Technologies, Inc., Aurora, IL, USA), leaf growth (number of leaves emerged since transplant), and leaf net photosynthetic rate via the CO_2_ exchange system LI-6400XT Portable Photosynthesis System (LI-COR, Nebraska, USA). For SPAD measurements, three leaves per plant were measured and the mean SPAD content was calculated per plant. For gas exchange measurements, three of five replicate plants per genotype within each soil origin were measured between 10 am-2 pm over a 2-day period to control for large diel differences in photosynthetic ally active radiation (PAR).

Individual plants were destructively harvested at 4 months after transplant. Likewise, bulk soil controls were destructively sampled. Each plant’s rooting system was subsampled for assessment of multiple response variables: root metabolomics for salicylate metabolite analysis, ectomycorrhizal root-tip colonization, and rhizosphere soils for 16S and ITS2 rRNA amplicon-based sequencing. Only fine-roots (< 2 mm diameter) were selected for these responses. For metabolomics, roots were quickly rinsed in DI water and frozen in liquid nitrogen immediately. For ectomycorrhizal colonization, a subset of roots was placed at 4 °C until analyzed (all samples were analyzed in ~ 1 month). Additional roots with attached rhizosphere soil and bulk soils were frozen at − 80 °C until processed for DNA extractions.

### Root metabolomic profiling and ectomycorrhizal colonization

Root tissues were analyzed using gas chromatography-mass spectrometry (GC-MS). Approximately 200 mg of fine roots were extracted in 2.5 ml of 80% ethanol twice. An aliquot of 1 ml of the combined extract was then dried in a nitrogen stream. Sorbitol was added to this mixture and used as an internal standard for relative metabolite quantification. The dried aliquot was dissolved in acetonitrile, followed by trimethylsilylation (TMS) for 2 days and then analyzed on GC-MS as described previously [55]. Metabolites were identified using the Wiley Registry 10th Edition with NIST 2014 mass spectral database and a large user-created database (~ 2400 TMS signatures). We explicitly chose genotypes based on higher-order salicylate profiles and thus targeted these specific metabolites for statistical analyses.

Additional roots for ectomycorrhizal colonization detection were rinsed in DI water, cut to 10 mm length, and randomly subsampled and viewed under a dissecting microscope. For each plant root sample, 100 root-tips were observed, and presence/absence of ECM scored to obtain the percentage of ECM root-tip colonization among each individual plant.

### DNA extractions and Illumina MiSeq sequencing preparation

All rhizosphere soils were washed from roots in 200 ml of sterile DI water and centrifuged at 10,000 rcf for 10 min and supernatant removed. Subsequently, genomic DNA was extracted from 250 mg of pelleted rhizosphere soil material using the MoBio PowerSoil DNA Isolation Kit (MoBio Laboratories, Inc., Carlsbad, CA) according to standard procedures except that extractions were lysed using a Precellys bead mill homogenizer (Life Science Products, Frederick, CO) at 5500 rpm for 3 cycles of 30 s bead-beat, 30 s rest. All extractions were quantified on a NanoDrop 1000 spectrophotometer (NanoDrop Products, Wilmington, DE) and quantities confirmed using the Qubit dsDNA Broad-Range assay (Thermo Scientific, USA) prior to PCRs.

A two-step PCR approach using frameshifting nucleotide primers was used for sequencing [56, 57] with barcode tagged reverse primers. Primers for bacterial PCRs included 8 forward and 6 reverse 515F/806R primers for the V4 region and 11 forward and 6 reverse primers for fungal ITS2 at equal molar concentrations (0.5 μM) [56]. Thermal cycler conditions for primary PCRs consisted of 5 cycles at 95 °C for 1 min, 50 °C for 2 min, and 72 °C for 1 min. Secondary PCRs consisted of denaturation at 95 °C for 45 s followed by 32 cycles of 95 °C for 15 s, 60 °C for 30 s, 72 °C for 30 s, and final extension at 72 °C for 30 s. Experimental units were pooled based on gel band intensity and then purified using Agencourt AMPure XP beads system (0.7:1 ratio; Beckman Coulter Inc., Pasadena, CA). Subsequently, Illumina MiSeq sequencing (v. 2; 2 × 250 cycles) were carried out using a 9pM amplicon concentration with a 15% PhiX spike.

### Bioinformatics processing

Before sequence processing, frameshift primers were removed using the cutadapt program in paired end legacy mode [58]. Next, paired-end sequences (.fastq) were processed using QIIME 1 [59]. Specifically, sequences were joined and demultiplexed using QIIME default settings, except using a Phred quality threshold of Q20. After demultiplexing, chimeras were screened using the QIIME-implemented UCHIME algorithm [60]. Detected chimeras were removed from .fasta files and then Operational Taxonomic Units (OTUs) were clustered at 97% similarity using the open reference workflow implementing UCLUST [61]. Only PyNAST-aligned OTU tables, without singletons, were used for bacterial community analyses. Bacterial OTUs were classified using RDP with the greengenes database (version 13.8) [62, 63] and fungal OTUs were classified using BLAST with the UNITE reference [64, 65]. Potential contaminant or artifact sequences (defined as unclassified at domain (archaea/bacteria) or kingdom (fungi) level, mitochondria, chloroplasts, plants or protista) were filtered from the dataset. OTUs with an abundance of < 10 sequences were also filtered and removed. For alpha-diversity estimates, the dataset was then rarefied at 13,000 sequences for bacteria and 3000 for fungi resulting in 21,019 OTUs and 1,690,000 sequences for archaea/bacteria, and 3534 OTUs and 411,000 sequences for fungi. For taxon abundances, raw counts were retained and normalized appropriately for statistical tests (as noted below) to deal with the compositional nature of sequence data [66]. Observed OTU richness, inverse of Simpsons Diversity (1-D), and Simpsons Evenness (E) were iteratively calculated in QIIME 1.

### Statistical analyses

A two-way ANOVA model was performed for each plant secondary metabolite (salicin, salicortin, α-salicyloylsalicin, salicylic acid, tremuloidin, trichocarpin, populin, catechin, total phenolics, total salicylates), trait measurement (photosynthetic rate, leaf chlorophyll content, leaf growth) and ectomycorrhizal root-tip colonization rates with soil origin and genotype as explanatory variables. If genotype was deemed a statistically significant predictor, a Tukey HSD post-hoc pairwise comparison test was performed. In addition, a multiple regression model with a stepwise selection and Akaike’s Information Criterion (AIC) minimization approach was performed to determine if plant responses and ectomycorrhizal root-tip colonization correlated with root secondary metabolite profiles. We also calculated variation inflation factors for regression models (*vif* function in package car in R) [67] and found that several metabolites exhibited multicollinearity (vif > 10; salicortin, α-salicyloylsalicin, total salicylates, and trichocarpin), and thus were not included in these final regression models. All plant responses were significantly greater in Clatskanie compared to Corvallis soils (Additional file 1: Table S3), therefore this multiple regression approach was performed for soil origin data separately. The majority of secondary metabolites were highly skewed thus were log_10_-transformed prior to regression analyses.

Microbial alpha-diversity and evenness also had a two-way ANOVA model performed with soil origin and genotype as explanatory variables. Further, a multiple regression model with stepwise selection and AIC minimization was also performed with secondary metabolites as explanatory variables. Soil origin was also included in these regression models to account for its overall large influence on microbial diversity estimates. For community composition, OTU-data was normalized using the centered log-ratio (clr) transformation (chemometrics package in R) [68] after a pseudocount of 1 was added to the data matrix to account for interdependence among samples and the “compositional” nature of sequence data [66]. Euclidean distances were calculated and implemented in a non-metric multidimensional scaling plot (NMDS) to visualize both archaeal/bacterial and fungal community compositional differences between genotypes and soil origin. A perMANOVA model (*adonis* in vegan package) [69] was also implemented to discern the amount of variation attributed to genotype, soil origin, and their interaction (with 999 permutations). In these analyses, soil origin was a stronger driver of compositional differences (Fig. 3), thus additional multivariate analyses were used with communities from Clatskanie and Corvallis soil origin separately. A constrained analysis of principal coordinates ([CAP], *capscale* function in vegan package) [69] was calculated for bacteria/archaea and fungi in Clatskanie and Corvallis soils with plant metabolites included as predictor variables. The CAP analysis had an additive constant added to correct for non-negative eigenvalues resulting from non-metric dissimilarities (add = T) [69]. Similar to NMDS analyses, Euclidean distances were calculated for CAP ordinations after a clr-transformation was applied to OTU counts. An ANOVA-like permutation test (999 permutations) was then used to determine if CAP models were deemed statistically significant and by which fixed effect terms (metabolites).

For microbial taxon abundances, raw counts of both bacterial/archaeal and fungal phyla and families were normalized by clr transformations [68]. Two-way ANOVAs were used to discern how soil origin, genotype, and their interactions influenced taxon abundances. If genotype was deemed a statistically significant predictor, a Tukey HSD post-hoc pairwise comparison test was performed to detect which specific genotypes differed. All Type 1 error rates had a Benjamini-Hochberg (FDR) *p*-value correction performed for running multiple ANOVA models at each taxonomic resolution (phyla, family models). Lastly, similar to plant trait variables, a stepwise regression model was used to understand how metabolites correlated with taxon abundances. Due to soil origin being a repeatedly strong predictor of taxa abundances, soil origin was also a predictor variable as well as metabolites for these models. All regression models within a taxonomic resolution had the Benjamini-Hochberg correction applied.

## Additional file

Additional file 1: **Table S1.** Soil characteristics for the two soil origins where soils were collected for the greenhouse study. **Table S2.** The mean (± 1 standard deviation) concentration of plant secondary metabolites (μg g-1 FW) across the 12 *Populus trichocarpa* genotypes grown in this study. GW-11032 had 3 samples grown in Corvallis soils that were destroyed and no data collected therefore only 2 replicates for this genotype in that soil origin are present across datasets. **Table S3.** Two-way ANOVA model summary for responses of plant measurements: photosynthetic rate (μmol m-2 s-1), leaf chlorophyll content, and leaf growth (no. since transplant) and explanatory variables of soil origin, genotype, and their interaction. Explanatory variables deemed statistically significant are bolded. **Table S4.** Stepwise regression model summary for responses of plant measurements: photosynthetic rate (μmol CO2 m-2 s-1), leaf chlorophyll content, and leaf growth (no. since transplant) and explanatory variables of salicylic acid and secondary metabolites. Only metabolites retained after AIC minimization for final model statistics are shown. **Table S5.** Two-way ANOVA results for dominant bacterial phyla (and class for Proteobacteria) and families with soil origin, genotype, and their interaction as explanatory variables. Taxon abundances were clr-transformed prior to ANOVAs. All Type-1 error rates were FDR-corrected. **Table S6.** Two-way ANOVA results for dominant fungal phyla and families with soil origin, genotype, and their interaction as explanatory variables. Taxon abundances were clr-transformed prior to ANOVAs. All Type-1 error rates were FDR-corrected. **Figure S1.** Bacterial and fungal diversity and evenness across genotypes and soil origins. Bacterial diversity and evenness and fungal evenness had a significant interaction among genotypes and soil origin (GxS) whereas fungal diversity did not differ among genotypes or soil origin. **Figure S2.** Constrained analysis of principal coordinates (CAP) plot visualizing rhizosphere bacterial/archaeal (Panel A, C) and fungal communities (Panel B, D) at the OTU-level across the 12 genotypes of study and within two differing soil origins (Clatskanie and Corvallis). Color denotes communities within different genotypes whereas circles denote Clatskanie soils and triangles denote Corvallis soils. (DOCX 703 kb)

## References

[1] Berendsen RL, Pieterse CMJ, Bakker PAHM (2012). The rhizosphere and plant microbiome health. Trends Plant Sci.

[2] Turner TR, James EK, Poole PS (2013). The plant microbiome. Genome Biol.

[3] Badri V, Chaparro JM, Zhang R, Shen Q, Vivanco JM (2013). Application of natural blends of phytochemicals derived from the root exudates of *Arabidopsis* to the soil reveal that phenolic-related compounds predominantly modulate the soil microbiome. J Biol Chemi.

[4] Bonito G, Reynolds H, Robeson MS, Nelson JA, Hodkinson BP, Tuskan G (2014). Plant host and soil origin influence fungal and bacterial assemblages in the roots of woody plants. Mol Ecol.

[5] Shakya Migun, Gottel Neil, Castro Hector, Yang Zamin K., Gunter Lee, Labbé Jessy, Muchero Wellington, Bonito Gregory, Vilgalys Rytas, Tuskan Gerald, Podar Mircea, Schadt Christopher W. (2013). A Multifactor Analysis of Fungal and Bacterial Community Structure in the Root Microbiome of Mature Populus deltoides Trees. PLoS ONE.

[6] Lundberg DS, Lebeis SL, Paredes SH, Yourstone S, Gehring J, Malfatti S (2014). Defining the core *Arabidopsis thaliana* root microbiome. Nature..

[7] Lebeis SL, Paredes SH, Lundberg DS, Breakfield N, Gehring J, McDonald M (2015). Salicylic acid modulates colonization of the root microbiome by specific bacterial taxa. Science..

[8] Wagner MR, Lundberg DS, del Rio TG, Tringe SG, Dangl JL, Mitchell-Olds T (2016). Host genotype and age shape the leaf and root microbiomes of a wild perennial plant. Nat Commun.

[9] Lareen A, Burton F, Schäfer P (2016). Plant root-microbe communication in shaping root microbiome. Plant Mol Biol.

[10] Fierer N., Jackson R. B. (2006). The diversity and biogeography of soil bacterial communities. Proceedings of the National Academy of Sciences.

[11] Kaiser K, Wemheuer B, Korolkow V, Wemheuer F, Heiko N, Schöning I, et al. Driving forces of soil bacterial community structure, diversity, and function in temperate grasslands and forests. Sci Rep. 2016. 10.1038/srep33696. 10.1038/srep33696 PMC5030646 27650273

[12] Toljander JF, Santos-Gonzalez JC, Tehler A, Finlay R (2008). Community analysis of arbuscular mycorrhizal fungi and bacteria in the maize mycorrhizophere in a long-term fertilization trial. FEMS Microbiol Ecol.

[13] Veach AM, Stokes CE, Knoepp J, Jumpponen A, Baird R (2018). Fungal communities and functional guilds shift along an elevational gradient in the southern Appalachian Mountains. Microb Ecol.

[14] Chau JF, Bagtzoglou AC, Willig MR (2011). The effect of soil texture on richness and diversity of bacterial communities. Environ Forensic.

[15] Mummey DL, Stahl PD (2004). Analysis of soil whole- and inner-microaggregate bacterial communities. Microb Ecol.

[16] Hansel CM, Fendorf S, Jardine PM, Francis CA (2008). Changes in bacterial and archaeal community structure and functional diversity along a geochemically variable soil profile. Appl Environ Microb.

[17] Garbeva P, Veen JA, Elsas JD (2004). Microbial diversity in soil: selection of microbial populations by plant and soil type and implications for disease suppressiveness. Annu Rev Phytopathol.

[18] Bulgarelli D, Rott M, Schlaeppi K (2012). Ver Loren van Themaat E, Ahmadinejad N, Assenza F, et al. revealing structure and assembly cues for *Arabidopsis* root-inhabiting bacterial microbiota. Nature..

[19] Bulgarelli D, Schlaeppi S, Spaepen V, Ver Loren van Themaat E, Schulze-Lefert P (2013). Structure and functions of the bacterial microbiota of plants. Annu Rev Plant Biol.

[20] Keddy PA (1992). Assembly and response rules: two goals for predictive community ecology. J Veg Sci.

[21] Bello F, Lavorel S, Lavergne S, Albert CH, Boulangeat I, Mazel F (2013). Hierarchical effects of environmental filters on the functional structure of plant communities: a case study in the French Alps. Ecography.

[22] Chaparro JM, Sheflin AM, Manter DK, Vivanco JM (2012). Manipulating the soil microbiome to increase soil health and plant fertility. Biol Fert Soils.

[23] Pál M, Kovác V, Szalai G, Soós V, Ma X, Liu H (2014). Salicylic acid and abiotic stress response in rice. J Agron Crop Sci.

[24] Khan MIR, Fatma M, Per TS, Anjum NA, Khan NA. Salicylic acid-induced abiotic stress tolerance and underlying mechanisms in plants. Front Plant Sci. 2015. 10.3389/fpls.2015.00462. 10.3389/fpls.2015.00462 PMC4485163 26175738

[25] Rivas-San Vicente M, Plasencia J (2011). Salicylic acid beyond defence: its role in plant growth and development. J Exp Bot.

[26] Miura K, Tada Y. Regulation of water, salinity, and cold stress responses by salicylic acid. Front Plant Sci. 2014. 10.3389/fpls.2014.00004. 10.3389/fpls.2014.00004 PMC3899523 24478784

[27] Malamy J, Carr JP, Klessig DF, Raskin I (1990). Salicylic acid: a likely exogenous signal in the resistance response of Tobacoo to viral infection. Science..

[28] Lee H, León J, Raskin I (1995). Biosynthesis and metabolism of salicylic acid. Proc Natl Acad Sci U S A.

[29] Kniskern JM, Traw MB, Bergelson J (2007). Salicylic acid and jasmonic acid signaling defense pathways reduce natural bacterial diversity on *Arabidopsis thaliana*. Mol Plant Microbe In..

[30] Doornbos RF, Geraats BPJ, Kuramae EE, Van Loon LC, Bakker PAHM (2011). Effects of jasmonic acid, ethylene, and salicylic acid signaling on the rhizosphere bacterial community of Arabidopsis thaliana. Mol Plant Microbe In.

[31] Silverman P, Seskar M, Kanter D, Schweizer P, Métraux J, Raskin I (1995). Salicylic acid in rice. Plant Physiol.

[32] Raskin I (1992). Role of salicylic acid in plants. Annu Rev Plant Phys.

[33] Koch JR, Creelman RA, Eshita SM, Seskar M, Mullet JE, Davis KR (2000). Ozone sensitivity in hybrid poplar correlates with insensitivity to both salicylic acid and jasmonic acid. The role of programmed cell death in lesion formation. Plant Physiol.

[34] Xue LJ, Guo W, Yuan Y, Anino EO, Nyamdari B, Wilson MC (2013). Constitutively elevated salicylic acid levels alter photosynthesis and oxidative state but not growth in transgenic *Populus*. Plant Cell.

[35] Donaldson JR, Stevens MT, Barnhill HR, Lindroth RL (2006). Age-related shifts in leaf chemistry of clonal aspen (Populus tremuloides). J Chem Ecol.

[36] Schweitzer JA, Madritch MD, Bailey JK, LeRoy CJ, Fischer DG (2008). From genes to ecosystems: the genetic basis of condensed tannins and their role in nutrient regulation in a *Populus* model system. Ecosystems..

[37] Schweitzer JA, Bailey JK, Rehill BJ, Martinsen GD, Hart SC, Lindroth RL (2004). Genetically based trait in a dominant tree affects ecosystem processes. Ecol Lett.

[38] Bailey JK, Deckert R, Schweitzer JA, Rehill BJ, Lindroth RL, Gehring C (2015). Host plant genetics affect hidden ecological players: links among Populus, condensed tannins, and fungal endophyte infection. Can J Botany.

[39] Schweitzer JA, Bailey JK, Bangert RK, Hart SC, Whitham TG, Bailey MJ, Lilley AK, Timms-Wilson TM, Spencer-Phillips PTN (2007). The role of plant genetics in determining above- and below-ground microbial communities. Microbial ecology of aerial plant surfaces.

[40] Schweitzer JA, Bailey JK, Fischer DG, LeRoy CL, Lonsdorf EV (2008). Plant-soil-microorganism interactions: heritable relationship between plant genotype and associated soil microorganisms. Ecology..

[41] Tuskan GA, Difazio S, Jansson S, Bohlmann J, Grigoriev I, Hellsten U (2006). The genome of black cottonwood, *Populus trichocarpa* (Torr. & gray). Science..

[42] Gottel NR, Castro HF, Kerley M, Yang Z, Pelletier DA, Podar M (2011). Distinct microbial communities within the endosphere and rhizosphere of *Populus deltoides* roots across contrasting soil types. Appl Environ Microb..

[43] Marschner P, Crowley D, Yang CH (2004). Development of specific rhizospheres bacterial communities in relation to plant species, nutrition and soil type. Plant Soil.

[44] Colin Y, Nicolitch O, Van Nostrand JD, Zhou JZ, Turpault MP, Uroz S. Taxonomic and functional shifts in the beech rhizosphere microbiome across a natural soil toposequence. Sci Rep. 2017. 10.1038/s41598-017-07639-1. 10.1038/s41598-017-07639-1 PMC5574896 28851878

[45] Sigurdssson BD, Thorgeirsson H, Linder S (2001). Growth and dry-matter partitioning of young *Populus trichocarpa* in response to carbon dioxide concentration and mineral nutrient availability. Tree Physiol.

[46] Kettler TA, Doran JW, Gilbert TL (2001). Simplified method for soil particle-size determination to accompany soil-quality analyses. Soil Sci Soc Am J.

[47] Eo J, Park KC, Kim MH (2015). Plant-specific effects of sunn hemp (Crotalaria juncea) and sudex (Sorghum bicolor x Sorghum biocolor var. sudanense) on the abundance and composition of soil microbial community. Agric Ecosyst Environ.

[48] Loake G, Grant M (2007). Salicylic acid in plant defence – the players and protagonists. Curr Opin Plant Biol.

[49] Zhalnina Kateryna, Louie Katherine B., Hao Zhao, Mansoori Nasim, da Rocha Ulisses Nunes, Shi Shengjing, Cho Heejung, Karaoz Ulas, Loqué Dominique, Bowen Benjamin P., Firestone Mary K., Northen Trent R., Brodie Eoin L. (2018). Dynamic root exudate chemistry and microbial substrate preferences drive patterns in rhizosphere microbial community assembly. Nature Microbiology.

[50] Mason CJ, Rubert-Nason KF, Lindroth RL, Raffa KF (2015). Aspen defense chemicals influence midgut bacterial community composition of gypsy moth. J Chem Ecol.

[51] Medina MJH, Gagnon H, Piché Y, Ocampo JA, Garrido JMG, Vierheilig H (2003). Root colonization by arbuscular mycorrhizal fungi is affected by the salicylic acid content of the plant. Plant Sci.

[52] Blilou I, Ocampo JA, Garrido JMG (1999). Resistance of pea roots to endomycorrhizal fungus or rhizobium correlated with enhanced levels of endogenous salicylic acid. J Exp Bot.

[53] Ansari A, Razmjoo J, Karimmojeni H (2016). Mycorrhizal colonization and seed treatment with salicylic acid to improve physiological traits and tolerance of flaxseed (*Linum usitatissimum* L.) plants grown under drought stress. Acta Physiol Plant.

[54] Pfabel C, Eckhardt KU, Baum C, Struck C, Frey P, Weih M (2012). Impact of ectomycorrhizal colonization and rust infection on the secondary metabolism of poplar (*Populus trichocarpa x deltoides*). Tree Physiol.

[55] Payyavula RS, Tschaplinski TJ, Jawdy SS, Sykes RW, Tuskan GA, Kalluri UC. Metabolic profiling reveals altered sugar and secondary metabolites in response to UGPase overexpression in Populus. BMC Plant Biol. 2014. 10.1186/s12870-014-0265-8. 10.1186/s12870-014-0265-8 PMC4197241 25287590

[56] Cregger MA, Veach AM, Yang ZK, Crouch MJ, Vilgalys R, Tuksan GA, et al. The *Populus* holobiont: dissecting the effects of plant niches and genotype on the microbiome. Microbiome. 2018. 10.1186/s40168-018-0413-8. 10.1186/s40168-018-0413-8 PMC5810025 29433554

[57] Lundberg DS, Yourstone S, Mieczkowski P, Jones CD, Dangl JL (2013). Practical innovations for high-throughput amplicon sequencing. Nat Methods.

[58] Martin M (2011). Cutadapt removes adapter sequences from high throughput sequencing reads. EMBnet J.

[59] Caporaso JG, Kuczynski J, Stombaugh J, Bittinger K, Bushman FD, Costello EK (2010). QIIME allows analysis of high-throughput community sequencing data. Nat Methods.

[60] Edgar RC, Haas BJ, Clemente JC, Quince C, Knight R (2011). UCHIME improves sensitivity and speed of chimera detection. Bioinformatics..

[61] Edgar RC (2010). Search and clustering orders of magnitude faster than BLAST. Bioinformatics..

[62] Wang Q, Garrity GM, Tiedje JM, Cole JR (2007). Naïve Bayesian classifier for rapid assignment of rRNA sequences into the new bacterial taxonomy. Appl Environ Microb..

[63] McDonald D, Price MN, Goodrich J, Nawrocki EP, DeSantis TZ, Probst A (2012). An improved Greengenes taxonomy with explicit ranks for ecological and evolutionary analyses of bacteria and archaea. ISME J.

[64] Altschul SF, Gish W, Miller W, Myers EW, Lipman D (1990). Basic local alignment search tool. J Mol Biol.

[65] Abarenkov K, Nilsson HR, Larsson KH, Alexander IJ, Eberhardt U, Erland S (2010). The UNITE database for molecular identification of fungi – recent updates and future perspectives. New Phytol.

[66] Gloor GB, Macklaim JM, Pawlowsky-Glahn V, Egozcue JJ (2017). Microbiome datasets are compositional and this is not optional. Front Microbiol.

[67] Venables WN, Riley BD (2013). Modern applied statistics with S.

[68] Wehrens R (2011). Chemometrics with R: multivariate data analysis in the natural sciences and life sciences.

[69] Oksanen J, Blanchet JG, Friendly M, Kindt R, Legendre P, McGlinn D, et al. Vegan: community ecology package. Ordination methods, diversity analysis and other function for community and vegetation ecologists: R package; 2018.

