# Correction to: Rhizosphere microbiomes diverge among *Populus trichocarpa* plant-host genotypes and chemotypes, but it depends on soil origin

**DOI:** 10.1186/s40168-021-01003-2

**Published:** 2021-01-22

**Authors:** Allison M. Veach, Reese Morris, Daniel Z. Yip, Zamin K. Yang, Nancy L. Engle, Melissa A. Cregger, Timothy J. Tschaplinski, Christopher W. Schadt

**Affiliations:** 1 Biosciences Division, Oak Ridge National Laboratory, 1 Bethel Valley Rd, Oak Ridge, TN 37831-6038 USA; 2 Department of Microbiology, University of Tennessee, Knoxville, TN 37996 USA

**Correction to: Microbiome 7, 76 (2019)**

**https://doi.org/10.1186/s40168-019-0668-8**

Following publication of the original article [1], the authors identified an error in Fig. 2. The correct figure is given below.

**Fig. 2:**
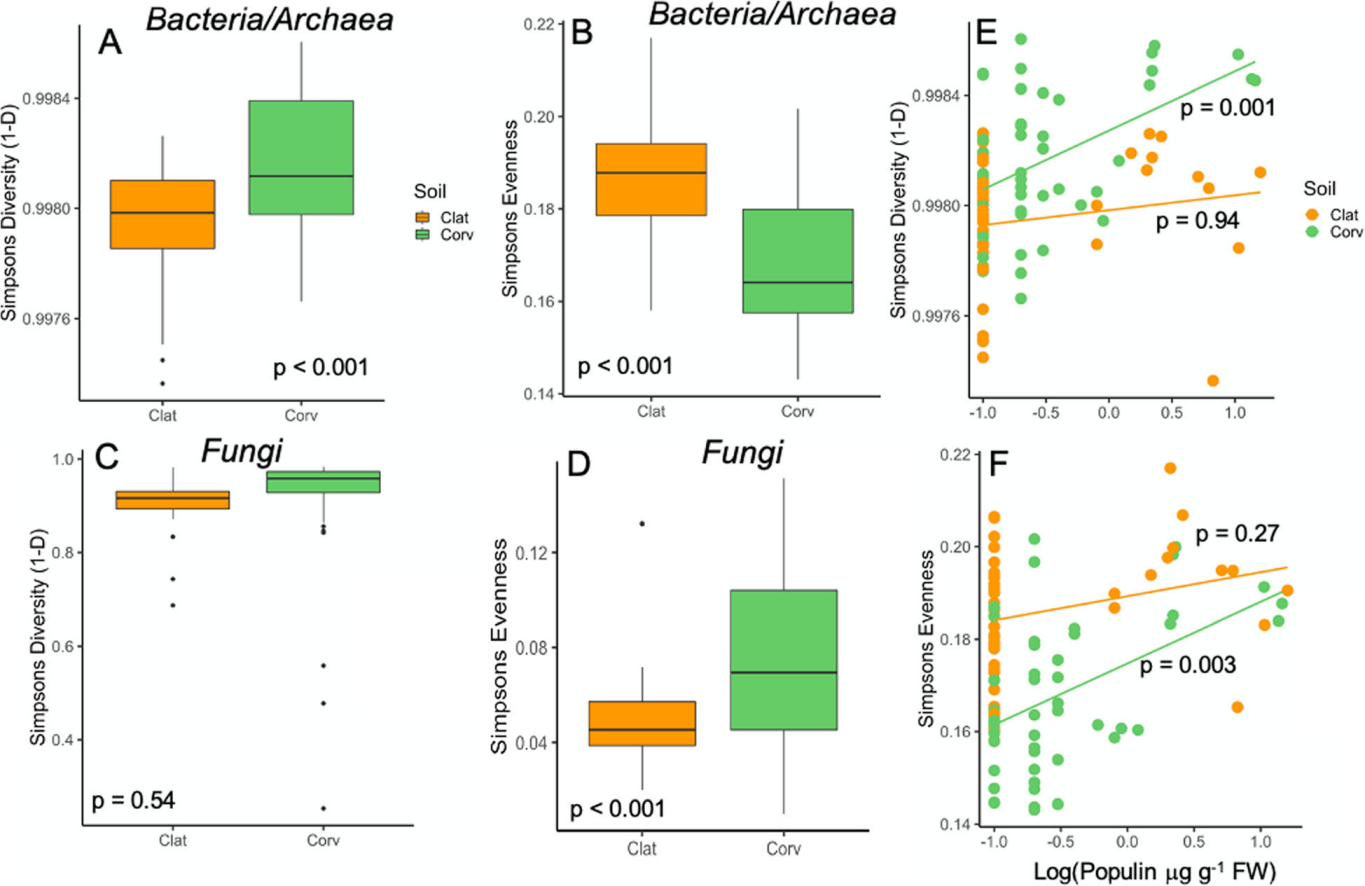
Bacterial/archaeal and fungal diversity (Simpson’s Diversity: 1-D; Panel **a**, **c**) and Simpsons’s Evenness (Panel **b**, **d**) in Clatskanie and Corvallis soil origins. Orange boxplots and points denote Clatskanie and green denotes Corvallis soils. Bacterial/archeal diversity and evenness was correlated with populin concentration in Corvallis soils (Panel **e**, **f**). Type-1 error rates given were generated by stepwise regression model analyses
