# Supplementary material for: Rhizosphere microbiomes diverge among Populus trichocarpa plant-host genotypes and chemotypes, but it depends on soil origin
Source: Microbiome. 2019 May 18;7:76. doi: 10.1186/s40168-019-0668-8 (PMC6525979; doi:10.1186/s40168-019-0668-8)

**Additional File 1**

**Table S1**. Soil characteristics for the two soil origins where soils were collected for the greenhouse study.

| **Soil Origin** | **GPS Coordinates** | **Soil Order** | **Soil Subgroup** | **% OM** | **%C** | **%N** | **% Sand** | **% Silt** | **%Clay** |
| --- | --- | --- | --- | --- | --- | --- | --- | --- | --- |
| Corvallis, OR | 44°35'17"N; 123°11'36"W | Mollisols | Fluventic Haploxerolls | 3.5 | 1.4 | 0.1 | 59.5 | 26.0 | 14.5 |
| Clatskanie, OR | 46°7'16"N; 123°16'11"W | Entisols | Typic Fluvaquents | 10.5 | 5.0 | 0.4 | 7.9 | 43.6 | 48.4 |

**Table S2**. The mean (± 1 standard deviation) concentration of plant secondary metabolites (μg g^-1^ FW) across the 12 *Populus trichocarpa* genotypes grown in this study. GW-11032 had 3 samples grown in Corvallis soils that were destroyed and no data collected therefore only 2 replicates for this genotype in that soil origin are present across datasets.

| **Genotype** | **total salicylates** | **total phenolics** | **salicylic**  **acid** | **a-salicyloylsalicin** | **salicin** | **salicortin** | **catechin** | **tremuloidin** | **trichocarpin** | **populin** |
| --- | --- | --- | --- | --- | --- | --- | --- | --- | --- | --- |
| BESC-289 | 9562 (1618) | 13137 (2041) | 414 (105) | 655 (159) | 547 (180) | 2980 (605) | 3575 (595) | 115 (50) | 4849 (781) | 0.1 (0.04) |
| HOMC | 9450 (2192) | 10839 (2253) | 838 (361) | 953 (262) | 387 (86) | 3173 (797) | 1389 (218) | 131 (30) | 3941 (860) | 0.1 (0.03) |
| KTMC | 9114 (1415) | 13270 (1144) | 404 (148) | 1049 (274) | 611 (120) | 3863 (712) | 4156 (653) | 84 (21) | 3088 (316) | 0.02 (0.01) |
| HOMA | 7821 (1728) | 9614 (1990) | 639 (287) | 929 (237) | 561 (116) | 3580 (852) | 1793 (349) | 132 (36) | 1979 (363) | 0.8 (0.3) |
| GW-9830 | 7588 (1979) | 10203 (1906) | 350 (153) | 692 (238) | 380 (91) | 2507 (821) | 2615 (277) | 94 (27) | 3563 (808) | 0.04 (0.02) |
| HARA | 7432 (1390) | 10393 (1713) | 393 (115) | 649 (150) | 309 (68) | 2901 (543) | 2961 (409) | 156 (49) | 3023 (607) | 0.02 (0.01) |
| GW-11032 | 7429 (1396) | 9707 (1595) | 488 (186) | 669 (177) | 529 (153) | 2259 (591) | 2279 (491) | 141 (39) | 3341 (553) | 0.1 (0.03) |
| BESC-395 | 6688 (1008) | 8666 (1147) | 401 (101) | 634 (152) | 411 (66) | 2254 (445) | 1978 (368) | 68 (11) | 2918 (526) | 0.02 (0.01) |
| BESC-194 | 5112 (992) | 7260 (1237) | 239 (91) | 482 (136) | 434 (98) | 1917 (437) | 2147 (409) | 74 (12) | 1946 (335) | 9.4 (1.6) |
| BESC-838 | 4601 (957) | 8535 (1241) | 129 (68) | 182 (66) | 282 (65) | 939 (483) | 3934 (652) | 64 (15) | 2992 (359) | 0.01 (0.01) |
| BESC-189 | 3920 (1453) | 5548 (1670) | 129 (90) | 286 (166) | 281 (68) | 1402 (682) | 1628 (574) | 99 (64) | 1719 (527) | 0.1 (0.03) |
| BESC-414 | 3376 (770) | 6442 (808) | 83 (26) | 186 (56) | 180 (42) | 903 (315) | 3066 (576) | 30 (8) | 1984 (390) | 1.9 (0.40) |

**Table S3**. Two-way ANOVA model summary for responses of plant measurements: photosynthetic rate (μmol m^-2^ s^-1^), leaf chlorophyll content, and leaf growth (no. since transplant) and explanatory variables of soil origin, genotype, and their interaction. Explanatory variables deemed statistically significant are bolded.

| **Response variable** | **Explanatory Variable** | **DF** | **F-value** | ***p*-value** |
| --- | --- | --- | --- | --- |
| Photosynthetic rate | **Soil Origin** | **1** | **15.55** | **<0.01** |
|  | **Genotype** | **11** | **2.47** | **0.02** |
|  | Interaction | 11 | 0.58 | 0.84 |
|  |  |  |  |  |
| Leaf chlorophyll | **Soil Origin** | **1** | **35.5** | **<0.01** |
|  | **Genotype** | **11** | **3.17** | **<0.01** |
|  | Interaction | 11 | 1.35 | 0.24 |
|  |  |  |  |  |
| Leaf growth | **Soil Origin** | **1** | **35.5** | **<0.01** |
|  | **Genotype** | **11** | **3.17** | **<0.01** |
|  | Interaction | 11 | 1.35 | 0.24 |

**Table S4**. Stepwise regression model summary for responses of plant measurements: photosynthetic rate (μmol CO_2_ m^-2^ s^-1^), leaf chlorophyll content, and leaf growth (no. since transplant) and explanatory variables of salicylic acid and secondary metabolites. Only metabolites retained after AIC minimization for final model statistics are shown.

|  |  |  |  |  | Full model statistics | | |
| --- | --- | --- | --- | --- | --- | --- | --- |
| Soil Origin | Response variable | Explanatory variable | T | *p* | F-statistic | Adj. R2 | *p*-value |
| Clatskanie | **Photosynthetic rate** | Intercept | 11.70 | *<0.01* | 3.83 | 0.18 | 0.04 |
|  |  | **catechin** | **-2.66** | **0.01** |  |  |  |
|  |  | phenolics | 1.70 | 0.10 |  |  |  |
|  |  |  |  |  |  |  |  |
|  | **Leaf chlorophyll content** | Intercept | 11.83 | <0.01 | 7.78 | 0.44 | <0.01 |
|  |  | **log(tremuloidin)** | **-4.25** | **<0.01** |  |  |  |
|  |  | catechin | -1.67 | 0.11 |  |  |  |
|  |  | **log(salicylic acid)** | **4.38** | **<0.01** |  |  |  |
|  |  |  |  |  |  |  |  |
|  | Leaf growth | Intercept | 2.68 | 0.01 | 3.25 | 0.15 | 0.06 |
|  |  | log(salicylic acid) | -2.40 | 0.02 |  |  |  |
|  |  | log(populin) | 1.84 | 0.08 |  |  |  |
|  |  |  |  |  |  |  |  |
| Corvallis | **Photosynthetic rate** | Intercept | 2.21 | 0.04 | 3.23 | 0.13 | 0.05 |
|  |  | **log(tremuloidin)** | **2.37** | **0.02** |  |  |  |
|  |  | phenolics | -1.85 | 0.08 |  |  |  |
|  |  |  |  |  |  |  |  |
|  | **Leaf chlorophyll content** | Intercept | 12.84 | <0.01 | 5.97 | 0.14 | 0.02 |
|  |  | **catechin** | **-2.44** | **0.02** |  |  |  |

**Table S5**. Two-way ANOVA results for dominant bacterial phyla (and class for Proteobacteria) and families with soil origin, genotype, and their interaction as explanatory variables. Taxon abundances were clr-transformed prior to ANOVAs. All Type-1 error rates were FDR-corrected.

| **Microbial Group** | **Explanatory Variable** | **DF** | **F-value** | ***p*-value** |
| --- | --- | --- | --- | --- |
| **Phylum** |  |  |  |  |
| *Actinobacteria* | Soil Origin | 1 | 76.06 | <0.01 |
|  | Genotype | 11 | 3.68 | <0.01 |
|  | Interaction | 11 | 1.82 | 0.2 |
| *Alphaproteobacteria* | Soil Origin | 1 | 20.89 | <0.01 |
|  | Genotype | 11 | 1.23 | 0.28 |
|  | Interaction | 11 | 1.58 | 0.28 |
| *Acidobacteria* | Soil Origin | 1 | 262.32 | <0.01 |
|  | Genotype | 11 | 9.32 | <0.01 |
|  | Interaction | 11 | 1.45 | 0.33 |
| *Betaproteobacteria* | Soil Origin | 1 | 29.37 | <0.01 |
|  | Genotype | 11 | 6.88 | <0.01 |
|  | Interaction | 11 | 1.86 | 0.2 |
| *Deltaproteobacteria* | Soil Origin | 1 | 71.15 | <0.01 |
|  | Genotype | 11 | 2.01 | 0.04 |
|  | Interaction | 11 | 2 | 0.2 |
| *Verrucocmicrobia* | Soil Origin | 1 | 98.79 | <0.01 |
|  | Genotype | 11 | 8.52 | <0.01 |
|  | Interaction | 11 | 0.96 | 0.54 |
| *Bacteroidetes* | Soil Origin | 1 | 46.03 | <0.01 |
|  | Genotype | 11 | 12.58 | <0.01 |
|  | Interaction | 11 | 0.76 | 0.68 |
| *Planctomycetes* | Soil Origin | 1 | 86.46 | <0.01 |
|  | Genotype | 11 | 13.36 | <0.01 |
|  | Interaction | 11 | 1.21 | 0.44 |
| *Chloroflexi* | Soil Origin | 1 | 106.1 | <0.01 |
|  | Genotype | 11 | 2.42 | 0.01 |
|  | Interaction | 11 | 2.08 | 0.2 |
| *Gammaproteobacteria* | Soil Origin | 1 | 101.94 | <0.01 |
|  | Genotype | 11 | 3.43 | <0.01 |
|  | Interaction | 11 | 1.04 | 0.51 |
| *Gemmatimonadetes* | Soil Origin | 1 | 5.71 | 0.02 |
|  | Genotype | 11 | 8.86 | <0.01 |
|  | Interaction | 11 | 1.4 | 0.33 |
| *Firmicutes* | Soil Origin | 1 | 7.13 | <0.01 |
|  | Genotype | 11 | 3.36 | 0.28 |
|  | Interaction | 11 | 1.15 | 0.28 |
| **Family** |  |  |  |  |
| *Hyphomicrobiaceae* | Soil Origin | 1 | 21.19 | <0.01 |
|  | Genotype | 11 | 2.22 | 0.03 |
|  | Interaction | 11 | 1.13 | 0.6 |
| *Gaiellaceae* | Soil Origin | 1 | 24.27 | <0.01 |
|  | Genotype | 11 | 2.51 | 0.01 |
|  | Interaction | 11 | 1.16 | 0.6 |
| *Chitinophagaceae* | Soil Origin | 1 | 52.84 | <0.01 |
|  | Genotype | 11 | 13.61 | <0.01 |
|  | Interaction | 11 | 0.78 | 0.71 |
| *Koribacteraceae* | Soil Origin | 1 | 1072.34 | <0.01 |
|  | Genotype | 11 | 14.34 | <0.01 |
|  | Interaction | 11 | 1.01 | 0.68 |
| *Comamonadaceae* | Soil Origin | 1 | 27.23 | <0.01 |
|  | Genotype | 11 | 4.35 | <0.01 |
|  | Interaction | 11 | 0.87 | 0.7 |
| *Chthoniobacteraceae* | Soil Origin | 1 | 5.02 | 0.03 |
|  | Genotype | 11 | 4.68 | <0.01 |
|  | Interaction | 11 | 2.03 | 0.16 |
| *Rhodospirillaceae* | Soil Origin | 1 | 171.26 | <0.01 |
|  | Genotype | 11 | 4.94 | <0.01 |
|  | Interaction | 11 | 0.93 | 0.68 |
| *Bradyrhizobiaceae* | Soil Origin | 1 | 12.2 | <0.01 |
|  | Genotype | 11 | 3.49 | <0.01 |
|  | Interaction | 11 | 1.61 | 0.32 |
| *Geobacteraceae* | Soil Origin | 1 | 34.998 | <0.01 |
|  | Genotype | 11 | 1.24 | 0.27 |
|  | Interaction | 11 | 1.93 | 0.17 |
| *Sinobacteraceae* | Soil Origin | 1 | 123.27 | <0.01 |
|  | Genotype | 11 | 6.13 | <0.01 |
|  | Interaction | 11 | 1.21 | 0.6 |
| *Gemmataceae* | Soil Origin | 1 | 109.71 | <0.01 |
|  | Genotype | 11 | 14.44 | <0.01 |
|  | Interaction | 11 | 1.15 | 0.6 |
| *Sphingomonadaceae* | Soil Origin | 1 | 111.6 | <0.01 |
|  | Genotype | 11 | 5.16 | <0.01 |
|  | Interaction | 11 | 2.47 | 0.16 |
| *Ellin515* | Soil Origin | 1 | 700.08 | <0.01 |
|  | Genotype | 11 | 16.42 | <0.01 |
|  | Interaction | 11 | 0.66 | 0.77 |
| *Solibacteraceae* | Soil Origin | 1 | 845.12 | <0.01 |
|  | Genotype | 11 | 14.19 | <0.01 |
|  | Interaction | 11 | 0.93 | 0.68 |
| *EB1003* | Soil Origin | 1 | 807.62 | <0.01 |
|  | Genotype | 11 | 7.78 | <0.01 |
|  | Interaction | 11 | 0.78 | 0.72 |
| *Xanthomonadaceae* | Soil Origin | 1 | 380.51 | <0.01 |
|  | Genotype | 11 | 8.04 | <0.01 |
|  | Interaction | 11 | 2.02 | 0.16 |
| *Rhodocyclaceae* | Soil Origin | 1 | 1.94 | 0.17 |
|  | Genotype | 11 | 2.13 | 0.03 |
|  | Interaction | 11 | 2.05 | 0.16 |

**Table S6.** Two-way ANOVA results for dominant fungal phyla and families with soil origin, genotype, and their interaction as explanatory variables. Taxon abundances were clr-transformed prior to ANOVAs. All Type-1 error rates were FDR-corrected.

| **Microbial Group** | **Explanatory Variable** | **DF** | **F-value** | ***p*-value** |
| --- | --- | --- | --- | --- |
| **Phylum** |  |  |  |  |
| *Ascomycota* | Soil Origin | 1 | 1.06 | 3.10E-01 |
|  | Genotype | 11 | 2.29 | 0.03 |
|  | Interaction | 11 | 1.64 | 0.11 |
| *Basidiomycota* | Soil Origin | 1 | 101.53 | <0.01 |
|  | Genotype | 11 | 0.89 | 0.55 |
|  | Interaction | 11 | 0.89 | 8.00E-02 |
| *Mortierellomycota* | Soil Origin | 1 | 5.13 | 3.00E-02 |
|  | Genotype | 11 | 7.07 | <0.01 |
|  | Interaction | 11 | 1.44 | 0.17 |
| *Glomeromycota* | Soil Origin | 1 | 7.16 | 0.02 |
|  | Genotype | 11 | 2.22 | 0.03 |
|  | Interaction | 11 | 0.67 | 0.76 |
| *Chytridiomycota* | Soil Origin | 1 | 11.77 | <0.01 |
|  | Genotype | 11 | 4.21 | <0.01 |
|  | Interaction | 11 | 1.2 | 0.3 |
| **Family** |  |  |  |  |
| *Pezizaceae* | Soil Origin | 1 | 24.98 | <0.01 |
|  | Genotype | 11 | 1.69 | 0.09 |
|  | Interaction | 11 | 1.62 | 0.18 |
| *Serendipitaceae* | Soil Origin | 1 | 168.57 | <0.01 |
|  | Genotype | 11 | 2.01 | 0.05 |
|  | Interaction | 11 | 1.73 | 0.18 |
| *Hydnangiaceae* | Soil Origin | 1 | 234.94 | <0.01 |
|  | Genotype | 11 | 3.28 | <0.01 |
|  | Interaction | 11 | 0.98 | 0.56 |
| *Hymenogastraceae* | Soil Origin | 1 | 209.54 | <0.01 |
|  | Genotype | 11 | 2.19 | 0.03 |
|  | Interaction | 11 | 1.88 | 0.18 |
| *Inocybaceae* | Soil Origin | 1 | 107.21 | <0.01 |
|  | Genotype | 11 | 2.83 | <0.01 |
|  | Interaction | 11 | 1.33 | 0.3 |
| *Piskurozymaceae* | Soil Origin | 1 | 131.27 | <0.01 |
|  | Genotype | 11 | 11.7 | <0.01 |
|  | Interaction | 11 | 1.64 | 0.18 |
|  |  |  |  |  |
| *Mortierellaceae* | Soil Origin | 1 | 3.74 | 0.07 |
|  | Genotype | 11 | 10.74 | <0.01 |
|  | Interaction | 11 | 2.02 | 0.18 |
| *Thelephoraceae* | Soil Origin | 1 | 57.16 | <0.01 |
|  | Genotype | 11 | 2.27 | 0.03 |
|  | Interaction | 11 | 1.81 | 0.18 |
| *Herpotrichiellaceae* | Soil Origin | 1 | 0.33 | 0.57 |
|  | Genotype | 11 | 3.34 | <0.01 |
|  | Interaction | 11 | 1.37 | 0.3 |
| *Aspergillaceae* | Soil Origin | 1 | 16.33 | <0.01 |
|  | Genotype | 11 | 4.18 | <0.01 |
|  | Interaction | 11 | 0.48 | 0.97 |
| *Nectriaceae* | Soil Origin | 1 | 6.11 | 0.02 |
|  | Genotype | 11 | 10.48 | <0.01 |
|  | Interaction | 11 | 1.9 | 0.18 |
| *Pyronemataceae* | Soil Origin | 1 | 2.31 | 0.15 |
|  | Genotype | 11 | 1.75 | 0.08 |
|  | Interaction | 11 | 0.36 | 0.97 |

**Table S7.**

|  |  |  |  | Full model statistics | | |
| --- | --- | --- | --- | --- | --- | --- |
| Microbial Family | Explanatory variable | T | *p-value* | F-statistic | Adj. R^2^ | *p*-value |
| **Bacteria** |  |  |  |  |  |  |
| *Chitinophagaceae* | Intercept | 33.46 | <0.01 | 11.56 | 0.24 | <0.01 |
|  | **Soil(Corvallis)** | **-3.57** | **<0.01** |  |  |  |
|  | **salicylic acid** | **-2.02** | **0.05** |  |  |  |
|  | **populin** | **2.47** | **0.02** |  |  |  |
|  |  |  |  |  |  |  |
| *Koribacteraceae* | Intercept | 14.19 | <0.01 | 144.2 | 0.85 | <0.01 |
|  | **Soil(Corvallis)** | **-20.08** | **<0.01** |  |  |  |
|  | catechin | 1.47 | 0.14 |  |  |  |
|  | **salicylic acid** | **-2.93** | **<0.01** |  |  |  |
|  | **populin** | **2.44** | **0.02** |  |  |  |
|  |  |  |  |  |  |  |
| *Comamonadaceae* | Intercept | 48.73 | <0.01 | 12.04 | 0.18 | <0.01 |
|  | **Soil(Corvallis)** | **-3.33** | **<0.01** |  |  |  |
|  | saliyclic acid | -1.88 | 0.06 |  |  |  |
|  |  |  |  |  |  |  |
| *Rhodospirillaceae* | Intercept | 48.64 | <0.01 | 45.77 | 0.57 | <0.01 |
|  | **Soil(Corvallis)** | **-9.69** | **<0.01** |  |  |  |
|  | salicylic acid | -1.5 | 0.14 |  |  |  |
|  | **populin** | **1.96** | **0.05** |  |  |  |
|  |  |  |  |  |  |  |
| *Geobacteraceae* | Intercept | 20.05 | <0.01 | 20.05 | 0.27 | <0.01 |
|  | **Soil(Corvallis)** | **-4.26** | **<0.01** |  |  |  |
|  | **salicylic acid** | **-2.48** | **0.02** |  |  |  |
|  |  |  |  |  |  |  |
| *Sphingomonadaceae* | Intercept | 9.65 | <0.01 | 18.6 | 0.46 | <0.01 |
|  | **Soil(Corvallis)** | **6.81** | **<0.01** |  |  |  |
|  | tremuloidin | -1.62 | 0.11 |  |  |  |
|  | **catechin** | **-2.52** | **0.01** |  |  |  |
|  | salicylic acid | -1.77 | 0.08 |  |  |  |
|  | **phenolics** | **2.16** | **0.03** |  |  |  |
|  |  |  |  |  |  |  |
| *Ellin515* | Intercept | 31.81 | <0.01 | 115.7 | 0.77 | <0.01 |
|  | **Soil(Corvallis)** | **-14.98** | **<0.01** |  |  |  |
|  | **salicylic acid** | **-3.23** | **<0.01** |  |  |  |
|  | **populin** | **2.95** | **<0.01** |  |  |  |
|  |  |  |  |  |  |  |
| *Solibacteraceae* | Intercept | 32.85 | <0.01 | 133.4 | 0.8 | <0.01 |
|  | **Soil(Corvallis)** | **-16.85** | **<0.01** |  |  |  |
|  | **salicylic acid** | **-2.25** | **0.03** |  |  |  |
|  | **populin** | **2.55** | **0.01** |  |  |  |
|  |  |  |  |  |  |  |
| *Rhodocylcaceae* | Intercept | 4.11 | <0.01 | 3.75 | 0.1 | <0.01 |
|  | **Soil(Corvallis)** | **2.41** | **0.02** |  |  |  |
|  | **catechin** | **2.26** | **0.03** |  |  |  |
|  | **salicylic acid** | **-2.83** | **<0.01** |  |  |  |
|  | populin | 1.55 | 0.12 |  |  |  |
| **Fungi** |  |  |  |  |  |  |
| *Hymenogastraceae* | Intercept | 15.32 | <0.01 | 101.1 | 0.65 | <0.01 |
|  | **Soil(Corvallis)** | **-13.67** | **<0.01** |  |  |  |
|  | **salicylic acid** | **2.46** | **0.02** |  |  |  |
|  |  |  |  |  |  |  |
| *Piskurozymaceae* | Intercept | 82.7 | <0.01 | 34.37 | 0.39 | <0.01 |
|  | **Soil(Corvallis)** | **-7.95** | **<0.01** |  |  |  |
|  | **populin** | **2.09** | **0.04** |  |  |  |
|  |  |  |  |  |  |  |
| *Mortierellaceae* | Intercept | 35.43 | <0.01 | 3.37 | 0.04 | 0.05 |
|  | **salicylic acid** | **-2.15** | **0.03** |  |  |  |
|  | populin | 1.49 | 0.14 |  |  |  |
|  |  |  |  |  |  |  |
| *Nectriaceae* | Intercept | 5.46 | <0.01 | 2.4 | 0.06 | 0.05 |
|  | **Soil(Corvallis)** | **-2.76** | **<0.01** |  |  |  |
|  | **tremuloidin** | **-2.05** | **0.04** |  |  |  |
|  | catechin | -1.75 | 0.08 |  |  |  |
|  | populin | 1.75 | 0.08 |  |  |  |
|  | phenolics | 1.89 | 0.06 |  |  |  |

**Figure S1.** Bacterial and fungal diversity and evenness across genotypes and soil origins. Bacterial diversity and evenness and fungal evenness had a significant interaction among genotypes and soil origin (GxS) whereas fungal diversity did not differ among genotypes or soil origin.


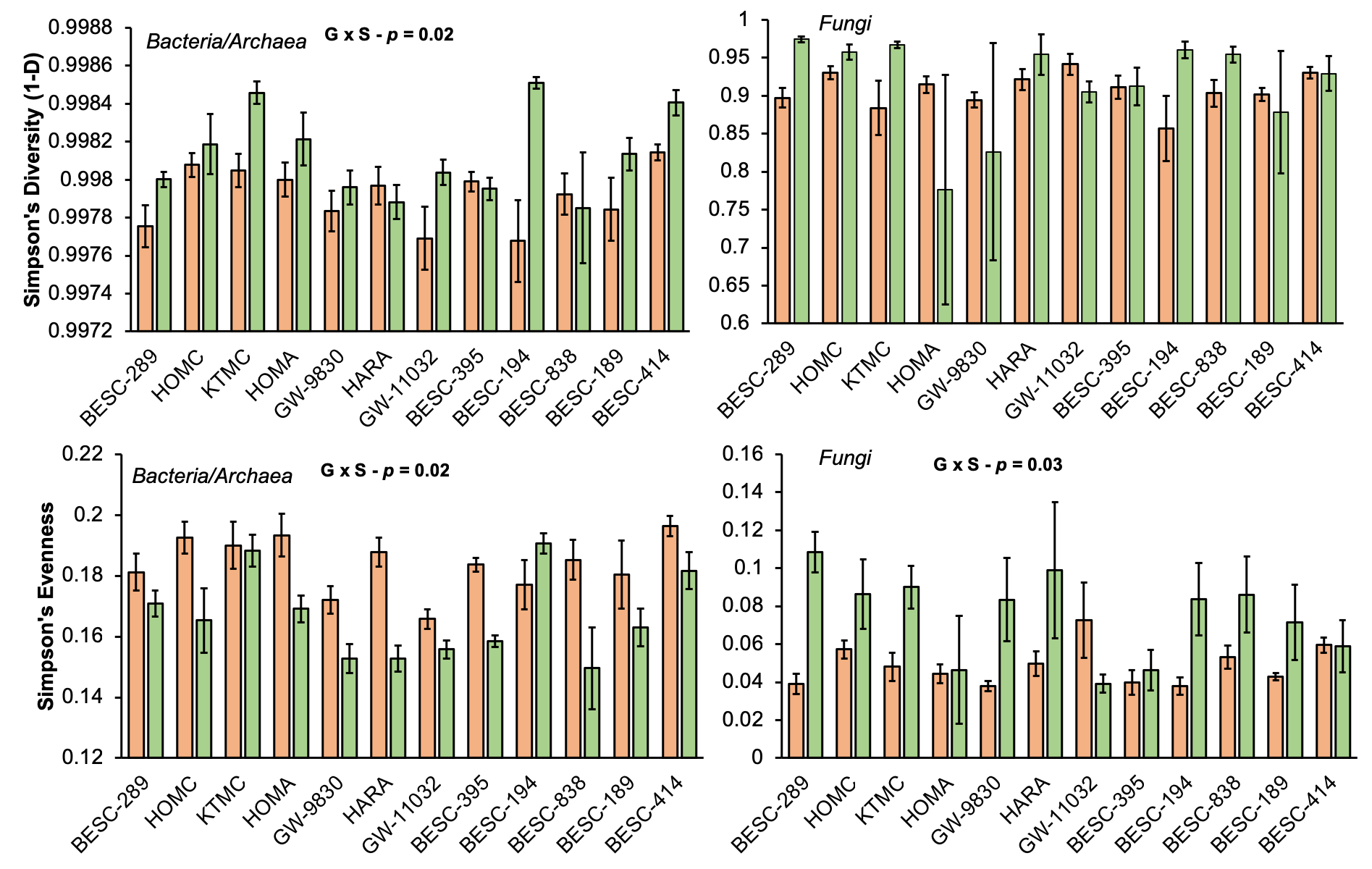


**Figure S2.** Constrained analysis of principal coordinates (CAP) plot visualizing rhizosphere bacterial/archaeal (Panel A, C) and fungal communities (Panel B, D) at the OTU-level across the 12 genotypes of study and within two differing soil origins (Clatskanie and Corvallis). Color denotes communities within different genotypes whereas circles denote Clatskanie soils and triangles denote Corvallis soils.


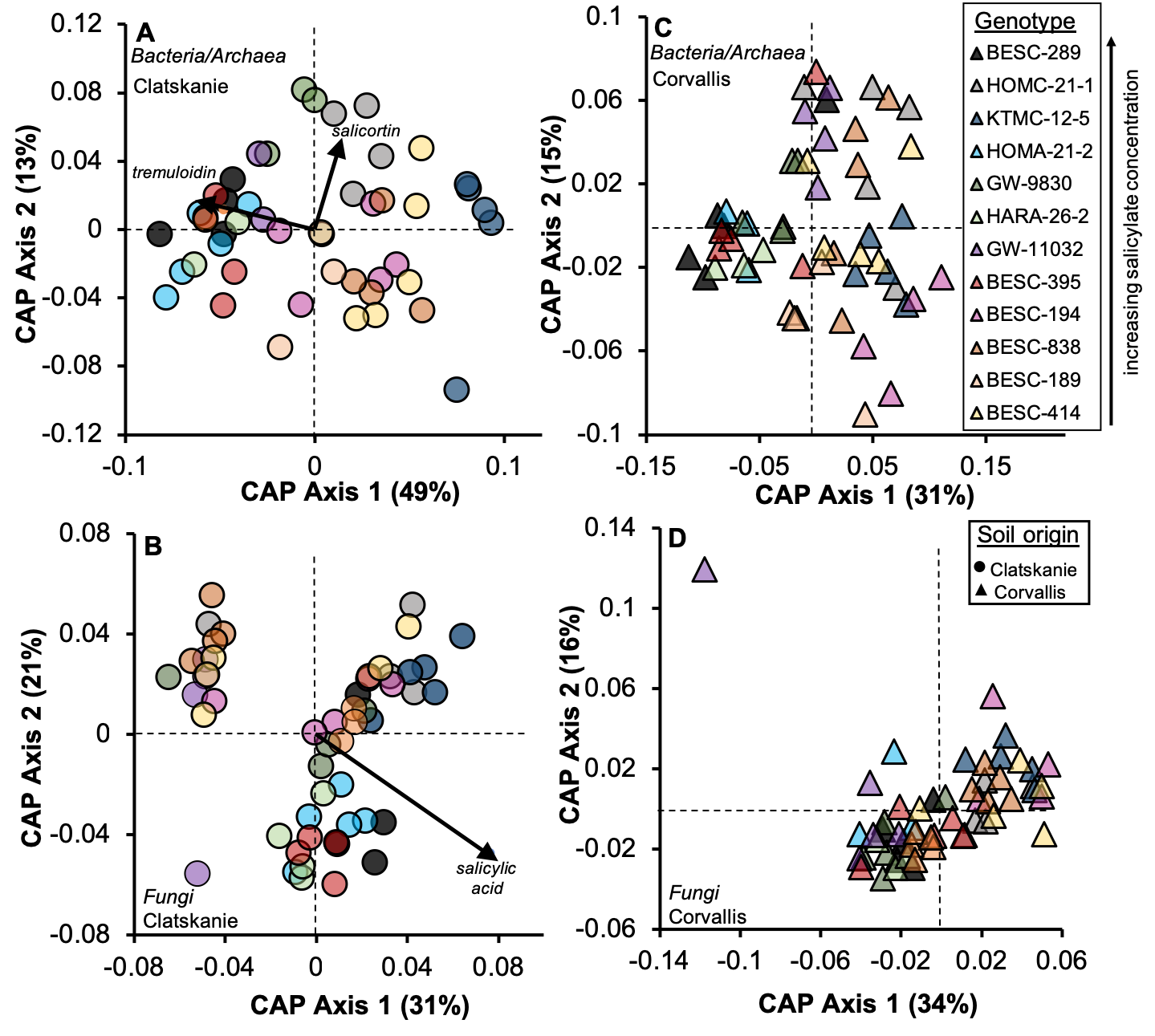

Supplement: Supplementary file 1 — Table S1. Soil characteristics for the two soil origins where soils were collected for the greenhouse study. Table S2. The mean (± 1 standard deviation) concentration of plant secondary metabolites (μg g-1 FW) across the 12 Populus trichocarpa genotypes grown in this study. GW-11032 had 3 samples grown in Corvallis soils that were destroyed and no data collected therefore only 2 replicates for this genotype in that soil origin are present across datasets. Table S3. Two-way ANOVA model summary for responses of plant measurements: photosynthetic rate (μmol m-2 s-1), leaf chlorophyll content, and leaf growth (no. since transplant) and explanatory variables of soil origin, genotype, and their interaction. Explanatory variables deemed statistically significant are bolded. Table S4. Stepwise regression model summary for responses of plant measurements: photosynthetic rate (μmol CO2 m-2 s-1), leaf chlorophyll content, and leaf growth (no. since transplant) and explanatory variables of salicylic acid and secondary metabolites. Only metabolites retained after AIC minimization for final model statistics are shown. Table S5. Two-way ANOVA results for dominant bacterial phyla (and class for Proteobacteria) and families with soil origin, genotype, and their interaction as explanatory variables. Taxon abundances were clr-transformed prior to ANOVAs. All Type-1 error rates were FDR-corrected. Table S6. Two-way ANOVA results for dominant fungal phyla and families with soil origin, genotype, and their interaction as explanatory variables. Taxon abundances were clr-transformed prior to ANOVAs. All Type-1 error rates were FDR-corrected. Figure S1. Bacterial and fungal diversity and evenness across genotypes and soil origins. Bacterial diversity and evenness and fungal evenness had a significant interaction among genotypes and soil origin (GxS) whereas fungal diversity did not differ among genotypes or soil origin. Figure S2. Constrained analysis of principal coordinates (CAP) plot vis [file 40168_2019_668_MOESM1_ESM.docx]
